# Altered Energy Homeostasis and Resistance to Diet-Induced Obesity in *KRAP*-Deficient Mice

**DOI:** 10.1371/journal.pone.0004240

**Published:** 2009-01-21

**Authors:** Takahiro Fujimoto, Kyoko Miyasaka, Midori Koyanagi, Toshiyuki Tsunoda, Iwai Baba, Keiko Doi, Minoru Ohta, Norihiro Kato, Takehiko Sasazuki, Senji Shirasawa

**Affiliations:** 1 Department of Cell Biology, Faculty of Medicine, Fukuoka University, Jonan-ku, Fukuoka, Japan; 2 Center for Advanced Molecular Medicine, Fukuoka University, Jonan-ku, Fukuoka, Japan; 3 Department of Clinical Physiology, Tokyo Metropolitan Institute of Gerontology, Tokyo, Japan; 4 Department of Gene Diagnostics and Therapeutics, Research Institute, International Medical Center of Japan, Shinjuku-ku, Tokyo, Japan; University of Parma, Italy

## Abstract

Obesity and related metabolic disorders have become leading causes of adult morbidity and mortality. KRAP (Ki-*ras*-induced actin-interacting protein) is a cytoskeleton-associated protein and a ubiquitous protein among tissues, originally identified as a cancer-related molecule, however, its physiological roles remain unknown. Here we demonstrate that *KRAP*-deficient (*KRAP^−/−^*) mice show enhanced metabolic rate, decreased adiposity, improved glucose tolerance, hypoinsulinemia and hypoleptinemia. *KRAP^−/−^* mice are also protected against high-fat diet-induced obesity and insulin resistance despite of hyperphagia. Notably, glucose uptake in the brown adipose tissue (BAT) in *KRAP^−/−^* mice is enhanced in an insulin-independent manner, suggesting that BAT is involved in altered energy homeostasis in *KRAP^−/−^* mice, although UCP (Uncoupling protein) expressions are not altered. Of interest is the down-regulation of fatty acid metabolism-related molecules, including acetyl-CoA carboxylase (ACC)-1, ACC-2 and fatty acid synthase in the liver of *KRAP* ^−/−^ mice, which could in part account for the metabolic phenotype in *KRAP^−/−^* mice. Thus, KRAP is a novel regulator in whole-body energy homeostasis and may be a therapeutic target in obesity and related diseases.

## Introduction

Obesity and related metabolic disorders have become leading causes of adult morbidity and mortality worldwide [1]. Obese individuals have an increased risk of insulin resistance, progressing to type 2 diabetes [2]–[4]. Increased adiposity develops when energy intake exceeds energy expenditure [5]. Understanding the regulatory circuits that govern energy, fat and glucose metabolism has been a major focus of research interest, and genetic manipulations of genes affecting these metabolisms revealed the importance of systemic intertissue communication for the underlying mechanisms [6]–[8].

*KRAP*, originally identified in a human colon cancer cell as a deregulated gene, is strongly expressed in liver, pancreas and brown adipose tissue (BAT) and rarely expressed in skeletal muscle or heart in the adult mouse [9], [10]. KRAP protein structure is highly conserved between human and mouse species (80% homology) and KRAP does not contain any known functional motifs predicted. No apparent paralogous gene with *KRAP* is found in these species. KRAP is immunohistologically localized along the bile canaliculi in a fashion of co-existence with filamentous-actin in mouse liver and is biochemically enriched in a cytoskeletal fraction, however, its physiological role has not been elucidated [10].

Here we established and characterized *KRAP* ^−/−^ mice, revealing that *KRAP* ^−/−^ mice displayed a profound metabolic phenotype that exhibits increased metabolic rate, reduced adiposity, improved glucose tolerance, hypoinsulinemia and hypoleptinemia. More importantly, *KRAP* ^−/−^ mice are protected against diet-induced weight gain, fatty liver formation and insulin resistance under a high-fat diet, implicating KRAP as a potential target in therapy for obesity and related diseases.

## Results

### *KRAP^−/−^* mice show altered energy homeostasis and decreased adiposity

**Figure 1:**
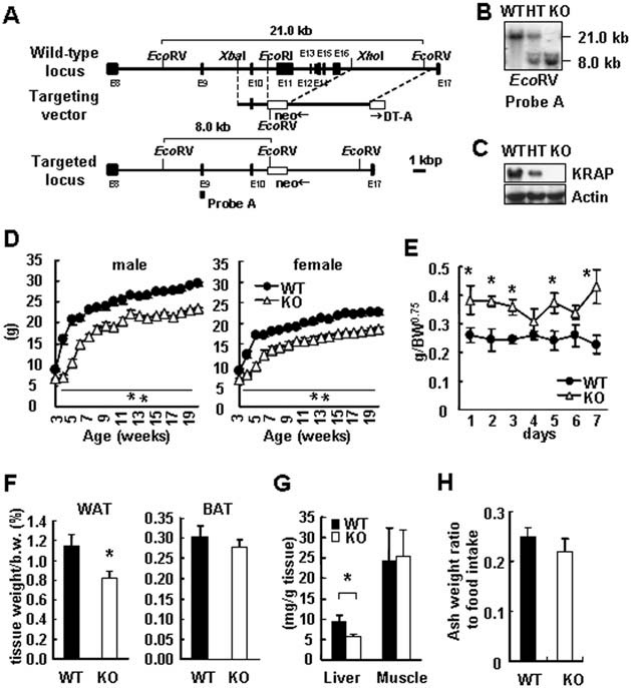
KRAP−/− Mice Showed Decreased Weight-Gain and Decreased Adiposity. (A) Targeted disruption of the KRAP gene. A region of the murine KRAP gene containing exons 11–16 was replaced with a neomycin resistance cassette. DT-A, Diphtheria-toxin A fragment; E, exon; neo, pGKneo. (B) Southern blotting of EcoRV digested tail DNA using the 5′ external probe A. WT, wild-type; HT, heterozygous; KO, KRAP−/−. (C) Western blotting with the KRAP antibody of liver lysates. (D) Body weight (BW) for KO and WT mice (n = 5–14 per group). P<0.01; F (1, 8) = 160.3 for male; P<0.01; F (1, 11) = 146.4 for female. (E) Food intake for KO and WT mice normalized by BW0.75. 30–36 weeks of age; n = 6. P<0.01; F (1, 10) = 12.6. (F) Percent body fat mass of epididymal white adipose tissue (WAT) and interscapular brown adipose tissue (BAT) (20 weeks of age; n = 9). (G) Tissue triglyceride content of livers and skeletal muscles, n = 9. (H) Daily ash mass of adult mice normalized to their food intake, n = 10. All the data are presented as mean±S.E.M.; *P<0.05; **P<0.01 compared with wild-type controls.

To examine the physiological role of KRAP, *KRAP* ^−/−^ mice were generated with deletion of 55% of the coding region of KRAP (Figure 1A). Homologous recombination was confirmed by Southern blotting (Figure 1B) and KRAP protein was not detected in *KRAP* ^−/−^-liver (Figure 1C). Genotype analysis of offspring from heterozygous matings was consistent with Mendelian inheritance (data not shown), suggesting that *KRAP* is dispensable in embryonic development. All the *KRAP* ^−/−^ mice in the mixed genetic background of 129SV/J and C57BL6/J were fertile and appeared to have a normal life span, however, *KRAP* ^−/−^ mice displayed a remarkable reduction in weight gain after birth (Figure S1). To further elucidate the physiological/pathological phenotypes, *KRAP* ^−/−^ mice with the genetic background of C57BL6/J were established and extensively analyzed.

Total body weight (BW) at birth was not different between *KRAP* ^−/−^ and wild-type mice (Table S1). *KRAP* ^−/−^ offspring appeared normal except for weight gain. A significant reduction in total BW was observed in *KRAP* ^−/−^ mice relative to wild-type littermates throughout life (Figure 1D). Although *KRAP* ^−/−^ mice displayed a significant increase in food intake (per BW^0.75^) (Figure 1E), their epididymal white adipose tissue (WAT) mass per BW was less than that of wild-type mice (Figure 1F). BAT mass in the *KRAP* ^−/−^ mice tended to be lower compared with wild-type mice, although the difference was not statistically significant (Figure 1F). Triglycerides in liver of *KRAP* ^−/−^ were less than those in the wild-type controls (Figure 1G). In contrast, neither muscle triglyceride contents nor liver glycogen contents were different between the genotypes (Figure 1G, Table S1).

The possibility that *KRAP* ^−/−^ mice show lean phenotype due to the result of poor nutrient absorption was examined. The ash weight ratio to food intake and the total amount of proteins in the feces were comparable between the genotypes (Figure 1H, Table S1). Furthermore, one-hour (h) postprandial blood glucose levels after overnight fasting were comparable between the genotypes (data not shown). These results show that malabsorption does not occur in the *KRAP* ^−/−^ mice.

Histological analysis of epididymal WAT and BAT showed a remarkable reduction in cell size (Figure S2), possibly due to the lower content of triglycerides in *KRAP* ^−/−^ mice. Furthermore, a reduction in the subcutaneous fat content of *KRAP* ^−/−^ mice was observed compared with that of wild-type mice (Figure S2). Liver and skeletal muscle of *KRAP* ^−/−^ mice had no obvious histological abnormality (Figure S2).

**Figure 2:**
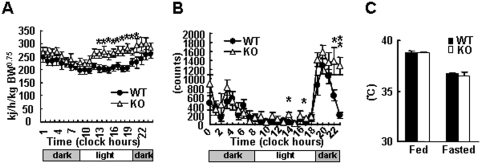
Enhanced Energy Expenditure in KRAP−/− Mice. (A) Whole-body energy expenditure measured by indirect calorimetry (22 weeks of age). 6,406±339 and 5,291±331 kJ/day/kg BW0.75), KRAP−/− (KO) and wild-type (WT) mice, respectively. P<0.05; F (1, 13) = 4.8; n = 7–8 per group. (B) Spontaneous ambulatory activity of mice evaluated for 24 h. Total ambulatory activity per day was 12,076±1,351 and 7,716±1,100 counts/day, KO and WT, respectively. P<0.05; F (1, 10) = 6.3; n = 6. (C) Rectal temperature in the fed or overnight-fasted conditions (n = 7–8). All the data are presented as mean±S.E.M.; *P<0.05; **P<0.01 compared with wild-type controls.

The phenotype of decreased weight gain despite normal food intake and absorption in *KRAP* ^−/−^ mice suggests that there must be an increase in whole-body energy expenditure. To explore this possibility, the metabolic rate was examined by indirect calorimetry, revealing that *KRAP* ^−/−^ mice showed an enhanced metabolic rate both in the daytime and at night (Figure 2A). Metabolic fuel source analyzed by respiratory quotient was comparable between the genotypes (Table S1). To characterize the mechanisms underlying higher metabolic rate in *KRAP* ^−/−^ mice, locomotor activity and rectal temperature were examined. *KRAP* ^−/−^ mice showed a slight increase in locomotor activity, which was more evident in the dark than in the light periods (Figure 2B). The slight increase in the locomotion of *KRAP* ^−/−^ mice seemed to reflect increased exploration for food, but the increase in locomotor activity partially account for the higher energy expenditure in *KRAP* ^−/−^ mice. On the other hand, rectal temperatures on fed or fasted conditions and UCP1 expression were not different between the genotypes (Figure 2C, S3). Furthermore, adaptive thermogenic response of the *KRAP* ^−/−^ mice to cold environment is not defective (data not shown), suggesting that thermogenesis may not account for the enhanced energy expenditure in *KRAP* ^−/−^ mice, although the possibility of enhanced heat radiation from the body surface of *KRAP* ^−/−^ mice has not been excluded.

### Protection against diet-induced obesity and insulin resistance in *KRAP^−/−^* mice

**Figure 3:**
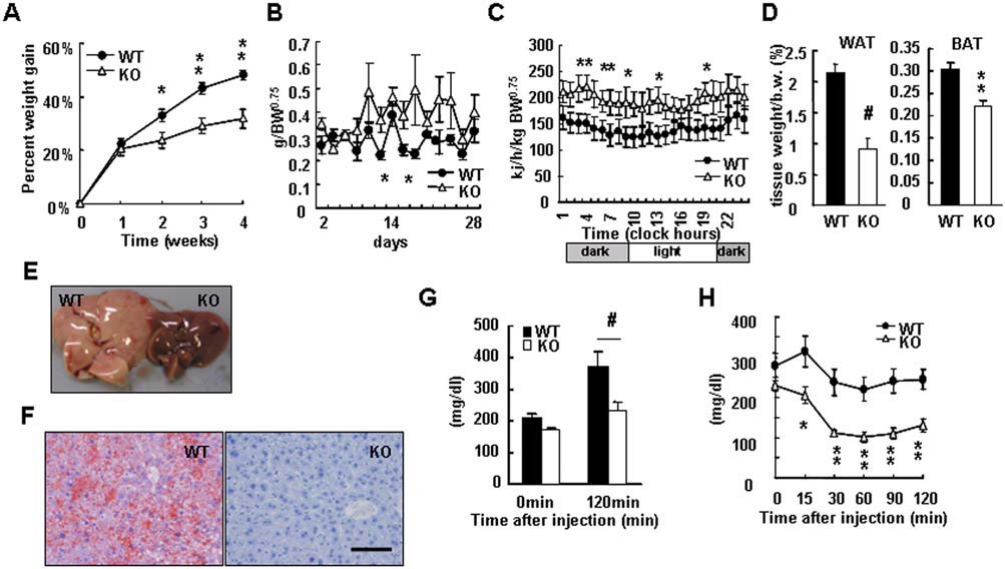
Protection Against Diet-Induced Obesity and Insulin Resistance in KRAP−/− Mice. (A) BW-gain under high-fat diet (HFD) for KRAP−/− (KO) and wild-type (WT) mice. n = 6–7; P<0.02; F (1, 11) = 9.5. (B) Food intake under HFD for KO and WT mice normalized by BW0.75. n = 6–7; P<0.05; F (1, 11) = 5.0. (C) Whole-body energy expenditure measured by indirect calorimetry after 4 weeks of an HFD. 4,694±598 and 3,389±496 kJ/day/kg BW0.75, KO and WT, respectively. n = 5–6; P<0.05; F (1, 9) = 5.9. (D) Percent body fat mass of epididymal white adipose (WAT) after 4 weeks of an HFD (left panel) or brown adipose tissue (BAT) after 18 weeks of an HFD (right panel). n = 6–7. (E) Gross appearance of liver from KO and WT mice after 14 weeks of an HFD. (F) Histological analysis of the livers represented in (E) performed by Oil Red-O and hematoxylin staining. Scale bar, 100 μm. (G) Blood glucose concentrations before and 120 min after the single glucose i.p. injection at a dose of 2 g glucose/kg BW. Glucose tolerance test performed after 8 weeks of an HFD (n = 7–8 per group). (H) Insulin sensitivity test performed after 8 weeks of an HFD. n = 7–8; P<0.01; F (1, 13) = 9.9. All the data are presented as mean±S.E.M.; *P<0.05; **P<0.01; #P<0.001 compared with wild-type controls.

Next, we asked whether these mice are protected against increased adiposity induced by a high-fat diet (HFD). When fed an HFD for 4 weeks, *KRAP* ^−/−^ and wild-type mice gained about 30% and 50% in BW, respectively (Figure 3A), although *KRAP* ^−/−^ mice displayed a significant increase in food intake (Figure 3B). The metabolic rate for *KRAP* ^−/−^ mice on an HFD was higher in comparison to wild-type levels (Figure 3C), whereas the respiratory quotient in *KRAP* ^−/−^ mice under HFD was comparable with that in the wild-type (Table S1). Epididymal WAT mass, BAT mass and tissue triglyceride contents in the liver and muscle were obviously decreased in *KRAP^−/−^* mice relative to the wild-type mice (Figure 3D, Table S1). Furthermore, *KRAP* ^−/−^ mice showed resistance to the formation of fatty liver (Figure 3E, 3F). These results together indicated that *KRAP* ^−/−^ mice do not become obese on an HFD.

We then asked whether *KRAP* ^−/−^ mice maintain insulin sensitivity on an HFD. Blood glucose levels tended to be lower in the *KRAP* ^−/−^ mice and basal insulin levels for *KRAP* ^−/−^ mice were lower than those for wild-type mice (Table S1). *KRAP* ^−/−^ mice also displayed excellent glucose tolerance and peripheral insulin action during HFD feeding (Figure 3G, 3H). All these results together suggested that *KRAP* ^−/−^ mice are protected against diet-induced obesity and insulin resistance.

### Serum glucose, insulin, leptin and triglycerides were lower in *KRAP* ^−/−^ mice

To more precisely understand the physiology of *KRAP* ^−/−^ mice, their serum chemistries were examined. Serum cholesterol, free fatty acid (FFA), ketone body, glucagon and albumin were indistinguishable between *KRAP* ^−/−^ and wild-type mice, whereas serum glucose, insulin, leptin and triglycerides were lower in *KRAP* ^−/−^ mice (Table S2). Adipokines such as leptin and adiponectin are thought to play an important role in the regulation of energy homeostasis and in glucose and fatty acid metabolism [11]–[14]. Serum leptin levels for *KRAP* ^−/−^ mice under HFD were also significantly lower than those of wild-type mice (Table S1). The hypoleptinemia can explain the hyperphagia, but not the enhanced energy expenditure in *KRAP* ^−/−^ mice [11]–[13]. On the other hand, serum adiponectin was higher in *KRAP* ^−/−^ mice under standard diet (Table S2), however, there was no difference under HFD between the genotypes (Table S1), suggesting that adiponectin may not be directly involved in the phenotype observed in *KRAP* ^−/−^ mice. There was no increase in thyroid hormones, T3 (triiodothyronine) or T4 (thyroxine), associated with an increase in metabolic rate. Growth hormone and IGF-1, implicated in maintaining body composition [15]–[17], were not significantly altered. These results together suggested that other factors and/or mechanisms will be involved in energy expenditure in *KRAP* ^−/−^ mice.

### ACC-1, ACC-2 and FAS were reduced in the *KRAP^−/−^*-liver

**Figure 4:**
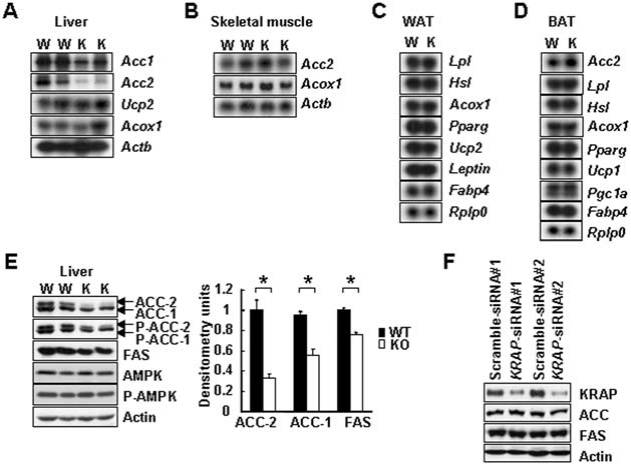
Reduced Expressions of ACC-1, ACC-2 and FAS in KRAP−/−-Liver. Northern blot analysis in liver (A), skeletal muscle (B), white adipose tissue (WAT; C) and brown adipose tissue (BAT; D) isolated from KRAP−/− (KO) and wild-type (WT) mice. (E) Western blot analysis of total-, phospho-ACC (P-ACC) and FAS of liver isolated from KO and WT mice (left). Densitometric quantification of ACC-1, ACC-2 and FAS normalized by actin content (right; n = 6; *P<0.001). Data are presented as mean±S.E.M. (F) Western blot analysis of ACC and FAS expressions in HepG2 cells 72 h after the transfection of siRNAs. Scramble-siRNAs were used as controls.

To understand molecular events in *KRAP* ^−/−^ mice, the expressions of mRNA involved in fatty acid metabolism, including *Acetyl-CoA carboxylase (Acc)*, *Acyl-CoA oxidase 1 (Acox1)*, *lipoprotein lipase (Lpl)*, *hormone sensitive lipase (Hsl)*, *peroxisome proliferators activated receptor gamma (Pparg)*, *Pparg coactivator 1 alpha (Pgc1a)*, *uncoupling protein (Ucp)*, *adipocyte fatty acid binding protein4 (Fabp4)* and *Leptin*, were examined in liver, skeletal muscle, WAT and BAT. Among these genes, *Acc2* is a critical regulator for the balance between synthesis and oxidation of fatty acid [18]. *Acc2*-deficient mice have a metabolic phenotype that exhibits increased metabolic rate and decreased adiposity [19], which is similar to that observed in *KRAP* ^−/−^ mice. Interestingly, *KRAP* ^−/−^ mice showed reduced expressions of *Acc1* and *Acc2* in the liver compared with the wild-type mice (Figure 4A), whereas the expression levels of *Acc2* in skeletal muscle and BAT did not differ between the genotypes (Figure 4B, 4D). *Ucp* is known to be involved in the regulation of thermogenesis and energy expenditure through the sympathetic nervous system [20], and *Acox1* catalyzes fatty acid oxidation. *KRAP*-deletion had no effect on the expressions of *Ucp* and *Acox1* in the tissues examined (*Ucp1* for BAT, Figure 4D; Figure S3; *Ucp2* for liver and WAT, Figure 4A and 4C; UCP-3 for skeletal muscle, Figure S4), which was consistent with no difference in rectal temperature measurements (Figure 2C). In WAT and BAT, the expressions of genes involved in fatty acid uptake, trapping and lipolysis, such as *Lpl*, *Fabp4*, *Hsl* and *Pparg* were not changed in *KRAP* ^−/−^ mice. Neither *Leptin* expression in WAT nor *Pgc1a* expression in BAT was different between the genotypes. All these results suggest that fatty acid synthesis is attenuated especially in the liver of *KRAP* ^−/−^ mice, where glucose oxidation and/or fatty acid oxidation might be inversely enhanced. Expressions of ACC-2, ACC-1 and fatty acid synthase (FAS) proteins were decreased by precisely 67%, 42% and 25% respectively in *KRAP* ^−/−^-liver (Figure 4E), which is consistent with the reduced mRNA levels and liver triglyceride content in *KRAP* ^−/−^ mice (Figure 4A, 1G).

To examine whether KRAP autonomously affects the expressions of lipogenic enzymes in cells, RNA interference-mediated suppression of KRAP in HepG2 cells was performed. No change in the levels of lipogenic genes was observed in the cells (Figure 4F), suggesting that KRAP may not autonomously regulate lipogenic gene expression in the cells. However, the possibility of the KRAP-autonomous effects in lipogenic gene expression is not excluded due to the incomplete knockdown of KRAP in this experiment.

From the viewpoint of ACC regulation, adiponectin is intriguing in that it phosphorylates and activates 5′-AMP-activated protein kinase (AMPK) [21], resulting in the phosphorylation and inactivation of ACC. However, the phosphorylation statuses of ACC-1, ACC-2 and AMPK were not different between the genotypes (Figure 4E), suggesting that the adiponectin-AMPK pathway is unlikely to contribute to the decreased expressions of the lipogenic genes in *KRAP* ^−/−^-liver. These results suggested that, although the precise pathway causing the attenuation of lipogenic enzymes in *KRAP* ^−/−^-liver remains unclear, the decrease in fatty acid synthesis-related genes including *Acc2* in *KRAP* ^−/−^-liver might function as a critical mediator of the altered energy homeostasis in *KRAP* ^−/−^ mice.

### Improved glucose tolerance and increased insulin-independent glucose uptake into *KRAP^−/−^* brown adipose tissue

**Figure 5:**
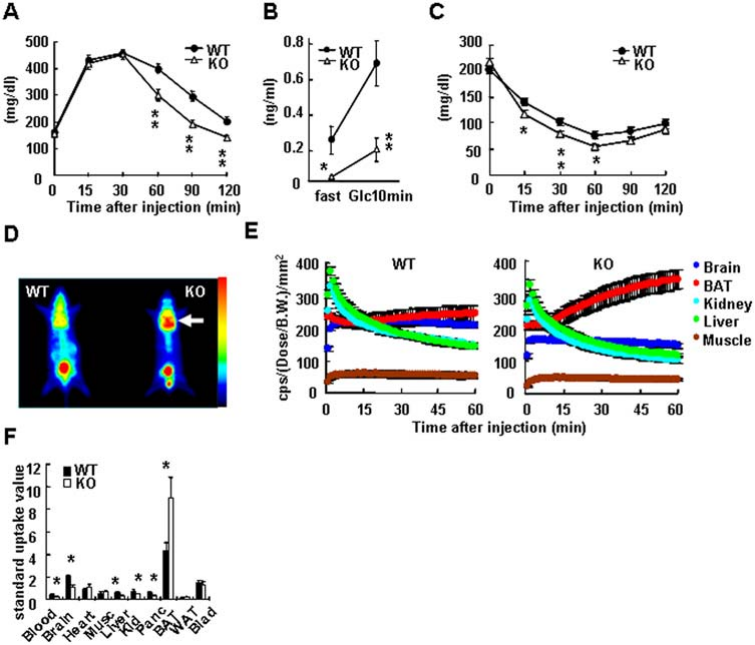
Improved Glucose Tolerance and Increased Insulin-Independent Glucose Uptake into KRAP−/− Brown Adipose Tissue. (A) Intraperitoneal glucose tolerance test in age-matched pair (15–17 weeks of age) of male mice on a standard diet. n = 10; P<0.01; F (1, 18) = 12.1. (B) Serum insulin concentrations before and 10 min after the single glucose i.p. injection at a dose of 2 g glucose/kg BW. Age-matched pair (9–11 weeks of age) of male mice. n = 8–10; P<0.01; F (1, 15) = 10.8. (C) Insulin sensitivity test in age-matched pair (21–27 weeks of age) of female mice on a standard diet. n = 14–15; P = 0.07; F (1, 27) = 3.5. (D) PET image showing the enhanced accumulation of 18F-FDG in the brown adipose tissue (BAT) of KRAP−/− (KO) mice. Arrow indicates a location of the BAT. A separate experiment in the same procedure was performed on five pairs of mice and the similar results were obtained. Gradation bar indicates signal intensity. (E) Time-activity curves of 18F-FDG in BAT (red circle), brain (dark blue circle), liver (green circle), kidney (pale blue circle) and muscle (brown circle) were obtained from the mean pixel radioactivity in ROI of the images shown in (D) (n = 5). (F) Quantification of the 18F-FDG uptakes in blood, brain, heart, muscle (Musc), liver, kidney (Kid), pancreas (Panc), BAT, epididymal white adipose tissue (WAT) and bladder (Blad) after the PET imaging (n = 5). All the data are presented as mean±S.E.M.; *P<0.05; **P<0.01 compared with wild-type controls.

As *KRAP* ^−/−^ mice show a significant reduction in circulating insulin levels with lower glucose levels (Table S2), a glucose tolerance test was performed. *KRAP* ^−/−^ mice displayed improved glucose tolerance with lower insulin levels compared with wild-type mice (Figure 5A, 5B), suggesting the possibility that peripheral insulin sensitivity and/or insulin-independent glucose uptake is enhanced in *KRAP* ^−/−^ mice.

Subsequently, an insulin sensitivity test was performed. The rate of glucose clearance upon insulin injection was slightly higher in *KRAP* ^−/−^ than in wild-type mice, suggesting that whole-body insulin sensitivity appears to be enhanced in the absence of *KRAP* (Figure 5C).

Next, to examine whether insulin-independent glucose uptake differs between the genotypes, the clearance of ^18^F-fluorodeoxyglucose (^18^F-FDG) was analyzed under an overnight-fasted condition by animal PET (positron emission tomography). PET imaging revealed the enhanced glucose uptake in BAT of *KRAP* ^−/−^ mice relative to wild-type mice (Figure 5D, 5E). Quantification of total glucose uptake into various tissues indicated the 2-fold higher uptake in *KRAP* ^−/−^-BAT compared with wild-type control (Figure 5F), whereas *KRAP* ^−/−^-BAT showed hypotrophic appearance (Figure 1F, S2, 3D). Besides BAT, there was no tissue showing higher glucose uptake in *KRAP* ^−/−^ mice relative to wild-type mice as far as we examined (Figure 5F). These results suggested that BAT plays a significant role in improved glucose tolerance and altered energy homeostasis in *KRAP* ^−/−^ mice. As the ^18^F-FDG dose used in the analysis had no effect on pancreatic insulin secretion, the higher glucose uptake in BAT of *KRAP* ^−/−^ mice is insulin-independent. Indeed, phosphorylation of Akt and insulin receptor were not enhanced in *KRAP* ^−/−^-BAT (Figure S5A, S5B), indicating that proximal insulin signaling is not involved in the higher glucose uptake in *KRAP* ^−/−^ mice and that the difference in insulin sensitivity may be a result due to relative higher insulin-independent glucose uptake in *KRAP* ^−/−^-BAT.

**Figure 6:**
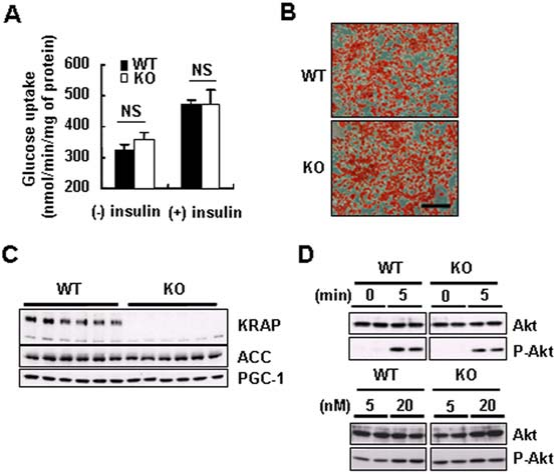
No Difference in Glucose Uptake, Differentiation or Insulin Signaling between KRAP−/−- and Wild-Type-Adipocytes in Vitro. (A) Glucose uptake into differentiated adipocytes, from KRAP−/− (KO)- and wild-type (WT)-BAT, measured with or without the pretreatment of insulin. One representative data of three independent experiments performed in quadruplicate. Data are presented as mean±S.E.M.; NS, not significant. (B) Accumulation of lipid droplets detected by Oil Red-O staining in the differentiated adipocytes. Scale bar, 100 μm. (C) Western blot analysis of ACC and PGC-1 in the differentiated adipocytes. (D) Western blot analysis of total- and phospho-Akt (P-Akt) in the differentiated adipocytes treated with 100 nM of insulin for the indicated period (upper) or with the indicated concentrations of insulin for 10 min (lower).

To examine whether the enhanced glucose uptake is autonomously caused by BAT cells, primary adipocytes from BAT were prepared. These showed that there was no significant difference in glucose uptake in the presence or absence of insulin between the genotypes (Figure 6A). Furthermore, *KRAP*-deletion in the cultured preadipocytes had no obvious effect on the differentiation process into adipocytes in histology and particular protein expressions (Figure 6B, 6C). Insulin-dependent Akt activation was examined, showing that the levels of phosphorylated Akt protein were comparable between the genotypes (Figure 6D). All these results suggested that not only cell autonomous effects of KRAP but also particular extracellular factors would be necessary for the improved glucose tolerance in *KRAP* ^−/−^-BAT and that KRAP in adipocytes would have a critical function in glucose uptake besides directly regulating insulin signaling.

## Discussion

*KRAP* ^−/−^ mice display a profound metabolic phenotype that exhibits higher basal metabolic rate, resulting in decreased adiposity concomitant with improved glucose tolerance, hypoinsulinemia and hypoleptinemia. *KRAP* ^−/−^ mice are protected against diet-induced obesity and insulin resistance under HFD. Tissue-specific insulin-independent glucose uptake in BAT will be involved in the metabolic phenotype in *KRAP* ^−/−^ mice.

Some of the *KRAP* ^−/−^ offspring in the C57BL6/J genetic background died during the nursing period, but not in the mixed genetic background of 129SV/J and C57BL6/J. The *KRAP* ^−/−^ offspring in the C57BL6/J genetic background exhibited a normal life span after weaning. The *KRAP* heterozygous mice do not exhibit the metabolic phenotype as far as we examined (data not shown), suggesting that sufficient reduction of KRAP expression beyond the particular threshold may be necessary for the manifestation in the whole-body metabolism. Taken together, abolition of *KRAP* does not severely influence adult life and KRAP could become a molecular target for lifestyle-related diseases.

The reduced fat deposits in *KRAP* ^−/−^ mice can be explained by the increased whole-body energy expenditure. As increased physical activity in *KRAP* ^−/−^ mice is restricted to dark period (Figure 2B), the higher physical activity does not fully account for the increased whole-body energy expenditure, suggesting that other processes such as heat production will be involved in the altered energy homeostasis in *KRAP* ^−/−^ mice. Although differences in rectal temperature and UCP-1 expression between the genotypes were not observed (Figure 2C, 4D, S3), the possibility of participation of heat radiation from the body surface of *KRAP* ^−/−^ mice in the higher metabolic rate is still not excluded. Another possibility is that fuel is consumed in other than heat production or fat storage in BAT [22], [23], because PET imaging confirmed that BAT is involved in the enhanced glucose uptake in *KRAP* ^−/−^ mice (Figure 5D–F).

Of note is the improved glucose tolerance in *KRAP* ^−/−^ mice. In general, leanness reveals increased insulin sensitivity and this will be a plausible idea applied for *KRAP* ^−/−^ mice (Figure 3H, 5C). Indeed, KRAP does not affect directly insulin signaling process as far as we examined (Figure 6D). On the other hand, our observation that *KRAP* ^−/−^-BAT predominantly uptake glucose in an insulin independent manner is interesting, and this finding will lead to new concepts for improved glucose metabolism. GLUT-1 is a candidate molecule involved in insulin-independent glucose uptake [24]. GLUT-1 expression levels in BAT are not different between the genotypes (Figure S3), suggesting that qualitative changes in GLUT-1 or the other transporters may contribute to the altered glucose uptake in *KRAP* ^−/−^-BAT. Elucidation of the molecular mechanisms beneath the insulin-independent glucose uptake in *KRAP* ^−/−^ mice may lead to the development of medicines for patients suffering from insulin resistance.

Recently, the importance of identification of networks rather than one or two genes has been reported to understand the molecular pathology of the diseases [25], [26]. Although incomplete KRAP knockdown should be considered in the HepG2-experiment, systemic intertissue communication might be involved in the metabolic phenotype of *KRAP* ^−/−^ mice, based on the finding that both the *in vitro* culture system of HepG2 and differentiated adipocytes do not reproduce phenomena observed in *KRAP* ^−/−^ mice *in vivo* (Figure 4F, 6A).

To discuss hypothetical molecular pathology underlying the metabolic phenotype of *KRAP* ^−/−^ mice, microarray gene expression analyses on BAT and liver were performed (Data were deposited in NCBI Gene Expression Omnibus. Accession number; GSE13585). To extract differentially expressed genes between the *KRAP* ^−/−^- and wild-type-tissues, Venn diagrams with 1.5-fold change in the three pairs comparisons was used as the filtering method, revealing that 272 probesets in BAT and 422 probesets in liver were changed in the three pairs of comparisons. Out of the 272 probesets, 114 and 158 were up- and down-regulated in *KRAP* ^−/−^-BAT, respectively (Figure S6). Out of the 422 probesets, 199 and 223 were up- and down-regulated in *KRAP* ^−/−^-liver, respectively (Figure S6). The down-regulated genes in *KRAP* ^−/−^-BAT were strongly associated with Gene Ontology (GO) terms such as “fatty acid biosynthesis” and “fatty acid metabolism” (Table S3), which is comparable with the appearance of a decreased adiposity in *KRAP* ^−/−^-BAT (Figure S2). Fatty acid desaturases such as *Scd1*, *Fads3* and *Sc5d* were found as the down-regulated genes in *KRAP* ^−/−^-BAT (Table S3). Among them, *Scd1* is remarkable from the viewpoint that *Scd1*-deficient mice show an increase in metabolic rate and a decrease in adiposity [27], [28], and *Scd1* might function as a critical mediator of the altered energy homeostasis in *KRAP* ^−/−^ mice.

Intriguing is that *Dio2*, which catalyzes the extrathyroidal production of the active thyroid hormone T3 via deiodination of T4 [22], is detected as one of the up-regulated genes in *KRAP* ^−/−^-BAT (Table S3). Although well-known critical regulators of energy homeostasis such as *Acox*, *Ppargc1a* (also known as *Pgc1a*) and *Ucp1* were not altered in our microarray analysis, the serum thyroid hormone level is low in *KRAP* ^−/−^ mice (Table S2), suggesting that the altered *Dio2* expression might be a molecular basis underlying the metabolic phenotype of the *KRAP* ^−/−^ mice. At this view point, the report that bile acids from liver affect the deiodinase activity in BAT [29] is fascinated, because KRAP protein localizes beneath the bile canalicular membrane of hepatocytes [10].

In *KRAP* ^−/−^-liver, the down-regulated genes were strongly associated with GO terms such as “cholesterol biosynthesis” and “lipid biosynthesis” (Table S4), which is consistent with the lower triglyceride contents in *KRAP* ^−/−^-liver (Figure 1G). Although some well-investigated lipogenic transcription factors including *Srebf1* were not altered, many critical regulators in lipid metabolism such as *Scd1*, *Acacb* (also known as *Acc2*), *Fasn* (also known as *Fas*), *Fabp5*, *Ppard*, *Acss2*, *Hmgcs1*, *Cyp51* and *Sc5d* were found as the down-regulated genes (Table S4, Figure 4A, 4E), suggesting the complexity of the molecular pathology of the *KRAP* ^−/−^ mice. Among the down-regulated genes, *Acc2* and *Scd1* are especially interesting, because both *Acc2*-deficient mice and *Scd1*-deficient mice show increased energy expenditure and decreased adiposity [19], [27], [28]. Considering the enhanced glucose uptake in *KRAP* ^−/−^-BAT, the down-regulation of fatty acid metabolism-related genes in *KRAP* ^−/−^-liver may result from the relatively lower glucose uptake into *KRAP* ^−/−^-liver (Figure 5E, 5F), however, the decrease in fatty acid metabolism-related genes including *Acc2* and *Scd1* in *KRAP* ^−/−^-liver might function as a critical mediator of the altered energy homeostasis in *KRAP* ^−/−^ mice.

Of interest is up-regulation of *Lepr* and *Acaa1a* (also known as *PTL*) in the *KRAP* ^−/−^-liver (Table S4), suggesting the presence of enhanced leptin sensitivity and its contribution to the metabolic phenotype of *KRAP* ^−/−^ mice [11]–[14]. In association with this observation, the discrepancy observed in *KRAP* ^−/−^ mice between decreased circulating serum leptin (Table S2) and normal WAT leptin mRNA levels (Figure 4) might suggest the existence of dysregulation of leptin production from WAT and/or increased peripheral leptin sensitivity in *KRAP* ^−/−^ mice. Based on the recent reports showing that insulin increases leptin translation [30], [31], hypoinsulinemia may affect the leptin turnover, which might contribute in part to the metabolic phenotypes in the *KRAP* ^−/−^ mice. As *Acaa1a* is suspected to be involved in fatty acyl-CoA β-oxidation pathway [32], assessment of the relevant pathways to the *KRAP* ^−/−^-liver metabolism should be a subject of future studies, although the expression level of *Acox* is not changed in the *KRAP* ^−/−^-liver (Figure 4A).

Although critical genes for the regulation of fatty acid metabolism and/or energy metabolism such as *Scd1*, *Acc2*, *Dio2* and *Lepr* were differentially expressed in *KRAP^−/−^*-BAT or liver, other multiple KRAP-regulated pathways and the interplays of these *in vivo* should be considered for potential mechanisms underlying the metabolic phenotypes in *KRAP* ^−/−^ mice. The smaller size of adipocytes in WAT and BAT may be one of the possible mechanisms protecting from lipotoxic effects of spilled fat to other tissues, which could also explain the improved glucose tolerance in the *KRAP* ^−/−^ mice. Another possibility of the participation of sympathetic nervous system is not excluded in the pathogenesis of *KRAP* ^−/−^ mice, although normal UCP1 levels and lower *Adrb3* levels existed in the *KRAP* ^−/−^ mice (Figure 4D, S3 and Table S3). Thus, the determination of the precise mechanism of the metabolic phenotype in *KRAP* ^−/−^ mice should await future studies.

In this study, we provide evidence to implicate a critical role for KRAP in pathways regulating whole-body energy homeostasis. Identification of the exact molecular functions of KRAP and tissue-specific conditional knockout mouse models will lead to a better understanding of the molecular basis for *KRAP*-deletion to affect whole-body energy homeostasis. Our work identifies KRAP as a promising new target for intervention in obesity and obesity-related diseases and establishes the *KRAP* ^−/−^ mouse as a novel model of metabolism-related diseases.

## Materials and Methods

### Animals

All the animals used in this study were treated in accordance with the rules of Fukuoka University.

### Generation of *KRAP*-deficient mice

We isolated a genomic DNA of the *KRAP* gene from a 129/SV mouse genomic library (Stratagene) and constructed the targeting vector by replacing a 7.2-kb *Xho*I-*Sal*I fragment containing exons 11–16 (55% of the coding region) of the *KRAP* gene, with a pGKneo cassette with opposite transcriptional orientation. The 5′ and 3′ arm of the targeting construct was composed of 2.3-kb and 6.4-kb genomic DNA, respectively. Diphtheria-toxin A fragment cassette (DT-A) flanked the 3′ genomic arm. The targeting vector was linearized with *Sal*I. ES cells were electroporated with the linearized targeting vector and selected with G418 on embryonic fibroblast feeder cells as described [33]. *Eco*RV digestion of genomic DNA identified an 8.0-kb recombinant allele and a 21.0-kb wild-type allele, when hybridized with an external probe (350-bp *Xba*I fragment). The neo probe was used to confirm homologous recombinants and ensure that there was only one integration site. The mutant ES cells were microinjected into C57BL6/J blastocysts as described [33], and the resulting male chimeras were mated with C57BL6/J mice. Heterozygous offspring were intercrossed to generate *KRAP*-deficient (*KRAP* ^−/−^) mice. The *KRAP* ^−/−^ line was backcrossed six times and maintained in the genetic background of C57BL6/J.

### BW regulation

Animals were maintained in a temperature-controlled (23°C) facility with a 12 h light/dark cycle. Mice had *ad libitum* access to water and standard rodent chow. Body weights were recorded weekly.

### Organ weight study

Age-matched (20 week-old) male *KRAP* ^−/−^ (*n* = 9) and wild-type (*n* = 9) mice were sacrificed and the wet weights of epididymal WAT and interscapular BAT were measured.

### Histological study

WAT, BAT, skin, liver and muscle of 22 week-old male mice were obtained under fed condition. Tissues were fixed in 3.7% formaldehyde in PBS, dehydrated in ethanol, embedded in paraffin. Sections were deparaffinized, rehydrated and stained with hematoxylin and eosin. Adipocyte perimeter from 200 cells was determined using Image J software from images of hematoxylin and eosin staining. Liver paraffin sections of mice after 14 weeks feeding of high-fat diet 32 (CLEA-Japan) were subjected to Oil Red-O staining to detect lipid droplets and counterstained with hematoxylin.

### Tissue triglyceride content

Tissue triglyceride was extracted [34], and measured with an enzymatic triglyceride E-test Wako (Wako Co.).

### Food intake study

Age-matched (30–36 week-old) male *KRAP* ^−/−^ and wild-type (*n* = 6) mice were given standard diet for 7 days. BWs were recorded every 24 h. Food, previously weighed, was offered to mice for 7 days. Every 24 h, the remaining food including crumbs was weighed and the daily food intake (g/day/BW^0.75^) was estimated.

### Energy metabolism study

Age-matched (22 week-old) male *KRAP* ^−/−^ (*n* = 8) and wild-type (*n* = 7) mice were kept for 3 days an individual metabolic cages (48 h for the acclimation of the mice to the metabolic cages, followed by 24 h for the measurement of the metabolic rate). Oxygen consumption and carbon dioxide production in expired air were measured continuously with an automatic O_2_-CO_2_ analyzer (NEC Medical System, model IH26) as described [35]. Energy expenditure per h and day was calculated.

### Locomotor activity and body temperature

Twenty four-h after the acclimation of the mice to the cages, spontaneous ambulatory activity of individually housed mice was evaluated over a 24 h period using a Digital Acquisition System (Bioresearch Center Co.). Consecutive photobeam brakes occurring in adjacent photobeams were scored as an ambulatory movement. Cumulative ambulatory activity counts were recorded every one h throughout the light/dark cycles. During the study, mice had *ad libitum* access to food and water. Body temperature was assessed by measuring rectal temperature using a digital thermometer (Shibaura Electronics Co.).

### BW regulation on a high-fat diet

Age-matched (23 week-old) male *KRAP* ^−/−^ (*n* = 6) and wild-type (*n* = 7) mice were given high-fat diet 32 (CLEA-Japan) for 4 weeks. BWs were recorded every 2 days. Food, previously weighed, was offered to mice for 4 weeks. Every 2 day periods, the remaining food, including crumbs, was weighed and the daily food intake (g/day/BW^0.75^) was estimated. After the 4 weeks period on a high fat diet, mice were subjected to measurements of energy metabolism as described above.

### Serum chemistry

Blood was collected from the retroorbital sinus in the fed state. Blood was kept on ice until centrifugation (3,000 g, 15 min at 4°C), and the serum was stored at −70°C until analysis. Serum insulin, leptin, adiponectin, T4, IGF-1 and glucagon concentrations were measured using ELISA kits (Shibayagi Co., R&D Systems, Otsuka Pharmaceutical Co., Endocrinetech. Co., R&D Systems and Yanaihara Research Co., respectively). Serum T3 and growth hormone were measured using RIA kits (Diagnostic Products Co. and GE Healthcare, respectively). Triglycerides, total cholesterol, FFA and albumin were determined by enzymatic assays (Wako Co.). Tail vein blood was used for glucose and ketone body quantifications with the Medisense Xtra (Abbott Laboratories Co.).

### Northern blotting

Northern blotting was performed as described [10]. The cDNA fragments of *Actb*, *Acox1*, *Acaca* (also known as *Acc1*), *Acacb* (also known as *Acc2*), *Ucp1*, *Ucp2*, *Lpl*, *Hsl*, *Pparg*, *Leptin*, *Fabp4*, *Rplp0* and *Pgc1a* were prepared by reverse transcriptase PCR using the primers listed in Table S5.

### Western blotting

Western blotting was performed as described [10]. Specific signals were detected by using KRAP antibody [10], Actin antibody (A2066, Sigma), phospho-ACC (Ser79) antibody (07-303, Upstate), Fatty acid synthase antibody (clone23, BD Biosciences), AMPK antibody (#2532, Cell Signaling), phospho-AMPK (Thr172) antibody (#2535, Cell Signaling), PGC-1α antibody (AB3242, Chemicon), Akt antibody (#9272, Cell Signaling) and Phospho-Akt (Ser473) antibody (#9271, Cell Signaling). To detect ACC proteins, avidin-HRP (GE Healthcare) was used.

### Cell culture

HepG2 cells were cultured at 37°C with 5% CO_2_ in DMEM containing 10% fetal calf serum (FCS), and transfected with a small inhibitory RNA (siRNA) using MicroPorator MP-100 (Digital Bio) as described [10]. Two distinct siRNAs were designed to target the coding region of human *KRAP* gene (nucleotides 1179–1203 and 1524–1548, GenBank accession no. **AB116937**) [10].

### Intraperitoneal glucose tolerance test, insulin secretion after glucose injection test or insulin sensitivity test

Intraperitoneal glucose tolerance test, using a dose of 2 g glucose/kg BW, was performed on overnight fasted animals. Insulin secretion after glucose injection test was carried out by measuring serum insulin concentrations before and 10 min after the single glucose i.p. injection at a dose of 2 g glucose/kg BW. Insulin sensitivity test was done on 3 h fasted animals followed by injection of insulin at a dose of 0.75 U/kg BW. Tail vein blood was collected 15, 30, 60, 90 and 120 min after injection and used for glucose quantification with the Medisense Xtra.

### Whole-body ^18^F-FDG PET

Age-matched (7-month-old) male wild-type (*n* = 5) and *KRAP* ^−/−^ (*n* = 5) mice were injected into a tail vein with 1 nmol/kg BW (150 MBq/kg BW) of 2-deoxy-2-[^18^F]fluoro-D-glucose (^18^F-FDG). The emission scan was started immediately after the injection and performed for 60 min with a planar positron imaging system as described [36]. At 65 min after the injection, mice were sacrificed and blood, brain, muscle, liver, kidney, pancreas, BAT, WAT and bladder were collected for ^18^F-FDG determination.

### Brown adipocyte cell culture

Brown adipocyte cell culture was done essentially as described [37]. Briefly, BAT was isolated from newborn *KRAP* ^−/−^ and wild-type mice, minced and subjected to collagenase digestion. The digested tissue was filtered, washed and the resultant cells were expanded in culture medium as described [37]. For differentiation, preadipocytes were grown to confluence in culture medium supplemented with 20 nM of insulin and 1 nM of T3 (differntiation medium). Confluent cells were incubated for 24 h in the differentiation medium further supplemented with 0.5 mM of isobutylmethylxanthine, 0.5 µM of dexamethasone and 0.125 mM of indomethacin. Subsequently, the cells were maintained in the differentiation medium for 5 days until exhibiting a fully differentiated phenotype with massive accumulation of fat droplets. Glucose uptake assay was carried out after the cells were starved in serum-free medium for 4 h, before 100 nM of insulin was added for another 30 min. At the end of the stimulation period, cells were exposed to 2-deoxy-[^3^H]glucose (0.5 µCi/ml final concentration) for 15 min. The incorporated radioactivity was determined by liquid scintillation counting.

### Expression array analysis

Total RNA was extracted from BAT or livers of three pairs of littermates (32 week-old) fed normal chow, using Qiagen RNeasy kit. Preparation of biotinylated antisnse cRNA, hybridization to GeneChip Mouse Genome 430 2.0 Array (Affymetrix), washing, scanning and data processing were performed according to the manufacturers' instructions and as described previously [10]. Output files were then loaded into GeneSpring v7.3 (Agilent Technologies) with per chip normalization to the 50th percentile and per-gene normalization to the average expression level of the wild-type. Three pairs of comparisons of the gene expression profile between *KRAP* ^−/−^ and the wild-type (KO#1 vs. WT#1, KO#2 vs. WT#2 and KO#3 vs. WT#3) was performed. To extract differentially expressed genes in KO compared with WT, Venn diagrams with 1.5-fold change in the three pairs comparisons was used as the filtering method.

### Statistical analysis

Data are presented as mean±S.E.M., and statistical analysis was performed using an unpaired Student's *t*-test when comparing two groups means (Figure 1F, 1G, 1H, 2C, 3D, 3G, 4E, 5F, 6A, S1, Table S1, S2). Data for the body weight, energy expenditure, respiratory quotient, locomotor activity, food intake on an HFD, insulin sensitivity, glucose tolerance and insulin secretion after glucose injection (Figure 1D, 1E, 2A, 2B, 3A, 3B, 3C, 3H, 5A, 5B, 5C) were subjected to repeated measures two-way ANOVA followed by *post hoc* Tukey-Kramer multiple comparisons tests. Data for the cell perimeter of white adipose tissue (Figure S2) was analyzed with Mann-Whitney U test. Differences at *P*<0.05 were considered to be statistically significant.

## Supporting Information

Figure S1 Body weight for KRAP−/− (KO) and wild-type (WT) mice. 18 week-old, Data are presented by mean±S.E.M. of n = 8.
(2.87 MB TIF)

Figure S2 Histological analysis of epididymal WAT, BAT, skin, liver and skeletal muscle of 22 week-old male mice by hematoxylin and eosin staining. Scale bar, 50 µm. (Right graph) The measurement of cell perimeter of epididymal white adipose tissue (WAT) by Image J software. Representative adipocyte perimeter distribution of 200 cells from two animals per group (Median, 193 µm for WT; 144 µm for KO. P<0.001 by Mann-Whitney U test).
(9.56 MB TIF)

Figure S3 No difference in expression levels of UCP-1, GLUT-1 or COX-4 in brown adipose tissue between KRAP−/− and wild-type mice. Interscapular brown adipose tissue (BAT) was obtained from KRAP−/− (KO) and wild-type (WT) mice. Protein expression levels of UCP-1, GLUT-1 and COX-4 were determined by western blotting. UCP-1 Ab, GLUT-1 Ab and COX-4 Ab were purchased from Sigma (U6382), Santa Cruz (H-43) and Clontech (S2207), respectively.
(1.94 MB TIF)

Figure S4 No difference in expression levels of UCP-3 or COX-4 in skeletal muscle between KRAP−/− and wild-type mice. Skeletal muscle was obtained from KRAP−/− (KO) and wild-type (WT) mice. Protein expression levels of UCP-3 and COX-4 were determined by western blotting. UCP-3 Ab, COX-4 Ab and Actin Ab were purchased from Sigma (U7757), Clontech (S2207) and Sigma (A2066), respectively.
(2.15 MB TIF)

Figure S5 Absence of significant activations of Akt or Insulin receptor β in the KRAP−/−-BAT in fasted condition. Interscapular brown adipose tissue (BAT) was obtained from KRAP−/− (KO) and wild-type (WT) mice. (A) Protein expression levels of Akt, phospho-Akt (Ser473) (P-Akt) and Actin were determined by western blotting. As a positive control for P-Akt, lysate from cultured adipocytes treated with 100 nM of insulin for 5 min was used. Akt Ab, phospho-Akt (Ser473) Ab and Actin Ab were purchased from Cell Signaling (#9272), Cell Signaling (#9271) and Sigma (A2066), respectively. (B) Protein expression levels of total- and phospho-Insulin receptor (IR) β (Tyr972) were determined by western blotting. Immunoprecipitates with IRβ Ab were immunoblotted by IRβ Ab (C-19, Santa Cruz) or phospho-IRβ (Tyr972) Ab (44-800G, BIOSOURCE).
(4.85 MB TIF)

Figure S6 Upper Venn diagrams showed the up-regulated 114 probesets and down-regulated 158 probesets in KRAP−/−-brown adipose tissue. Lower Venn diagrams showed the up-regulated 119 probesets and down-regulated 223 probesets in KRAP−/−-liver. A cut-off value of 1.5-fold or more change between the KRAP−/− (KO) and the wild-type (WT) tissues was used. The data of the differentially expressed genes were provided in Table S3 and S4.
(10.08 MB TIF)

Table S1 Metabolic profiles of KRAP−/− and wild-type mice in the C57BL6/J genetic background. Body weights of KRAP−/− and wild-type mice were measured at birth. For liver glycogen measurement, livers from 20 week-old male mice under standard diet were homogenized in 3% (w/w) perchloric acid and the homogenate was incubated for 2 h at 40°C with amyloglucosidase to hydrolyze the glycogen. The resulting glucose residue was then measured using an enzymatic glucose test Wako. The content of undigested proteins in fecal samples was examined using a Bradford ULTRA (novexin) with water-homogenized fecal samples. Oxygen consumption and carbon dioxide production in expired air were measured continuously with an automatic O2-CO2 analyzer (NEC Medical System, model IH26) and the respiratory quotient was calculated. Respiratory quotient of mice after 4 weeks of high-fat diet were also examined. Triglyceride content in liver and muscle from mice fed a high-fat diet for 14 weeks was measured with triglyceride E-test Wako (Wako Co.). Serum glucose was measured after 10 weeks of high-fat diet with the Medisense Xtra (Abbott Laboratories Co.). Serum insulin, leptin and adiponectin were measured after 10 weeks of high-fat diet with ELISA kits (Shibayagi Co., R&D Systems and Otsuka Pharmaceutical Co., respectively). n, number; Data are presented by mean±S.E.M.
(0.04 MB DOC)

Table S2 Serum chemistry of wild-type and KRAP−/− mice. All samples were from mice on a standard diet and assessed for serum parameters as described in Methods. Data are presented as the mean±S.E.M.; *P<0.05 and P<0.01 compared with wild-type controls. n, number; FFA, free fatty acid; T3, triiodothyronine; T4, thyroxine.
(0.05 MB DOC)

Table S3 Gene Ontology (GO) terms enriched in the down-regulated genes in KRAP−/−-BAT. Out of 158 down-regulated genes, 104 genes were found to have GO term annotations and subjected to GO term enrichment analysis. Expression data for 1.5-fold or more down- and up-regulated genes in KRAP−/−-BAT. Microarray gene expression analysis was performed on the brown adipose tissues from three pairs of KRAP−/− (KO) and the wild-type (WT) controls.
(0.64 MB DOC)

Table S4 Gene Ontology (GO) terms enriched in the down-regulated genes in KRAP−/−-liver. Out of 223 down-regulated genes, 151 genes were found to have GO term annotations and subjected to GO term enrichment analysis. Expression data for 1.5-fold or more down- and up-regulated genes in KRAP−/−-liver. Microarray gene expression analysis was performed on the livers from three pairs of KRAP−/− (KO) and the wild-type (WT) controls.
(0.95 MB DOC)

Table S5 Primer sequences for preparation of probes used in northern blotting.
(0.03 MB DOC)
