# Supplementary figures and images for: Altered Energy Homeostasis and Resistance to Diet-Induced Obesity in KRAP-Deficient Mice
Source: PLoS One. 2009 Jan 21;4(1):e4240. doi: 10.1371/journal.pone.0004240 (PMC2627767; doi:10.1371/journal.pone.0004240)

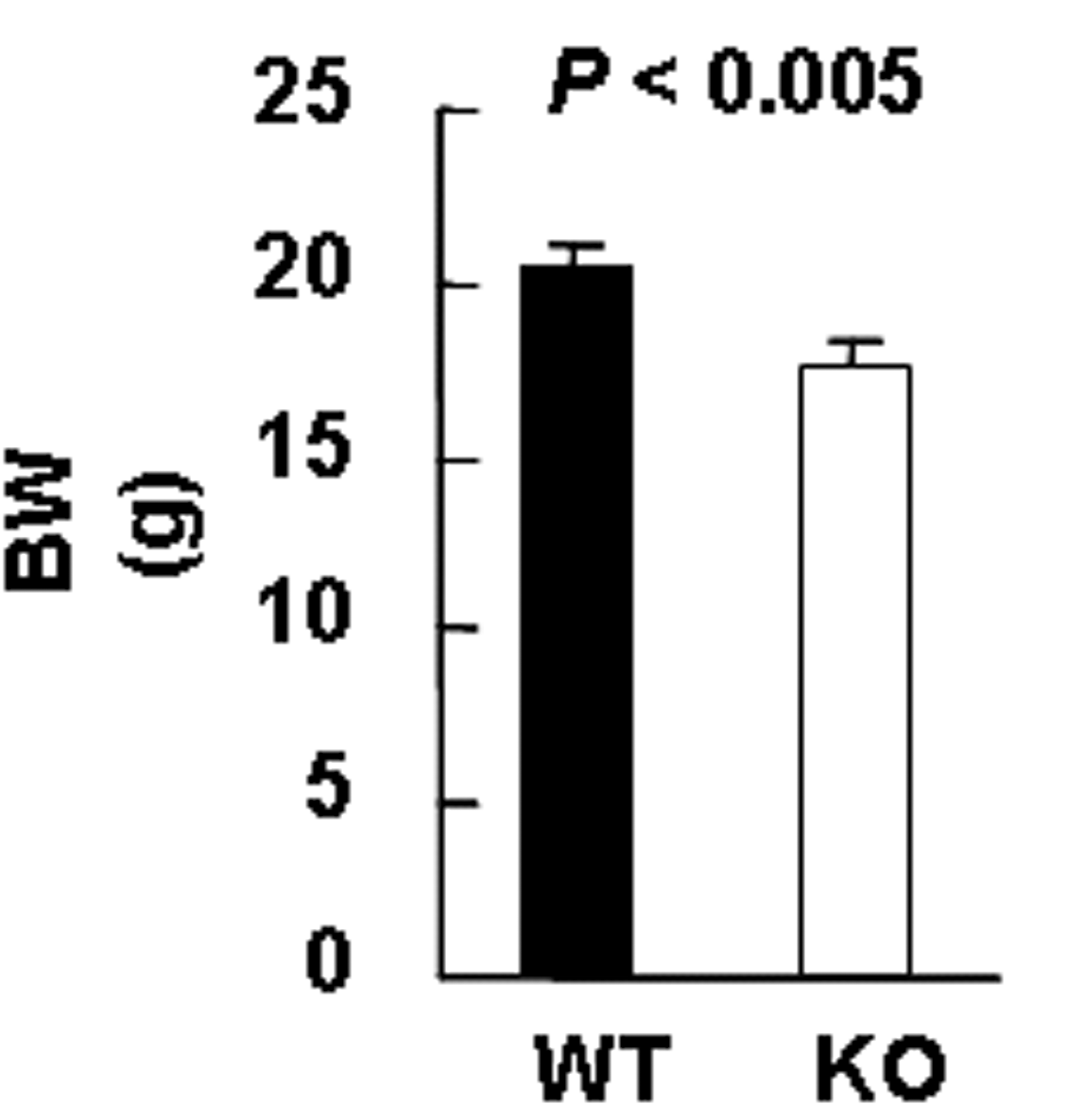

Supplement: Figure S1 — Body weight for KRAP−/− (KO) and wild-type (WT) mice. 18 week-old, Data are presented by mean±S.E.M. of n = 8. (2.87 MB TIF) [file pone.0004240.s001.tif]

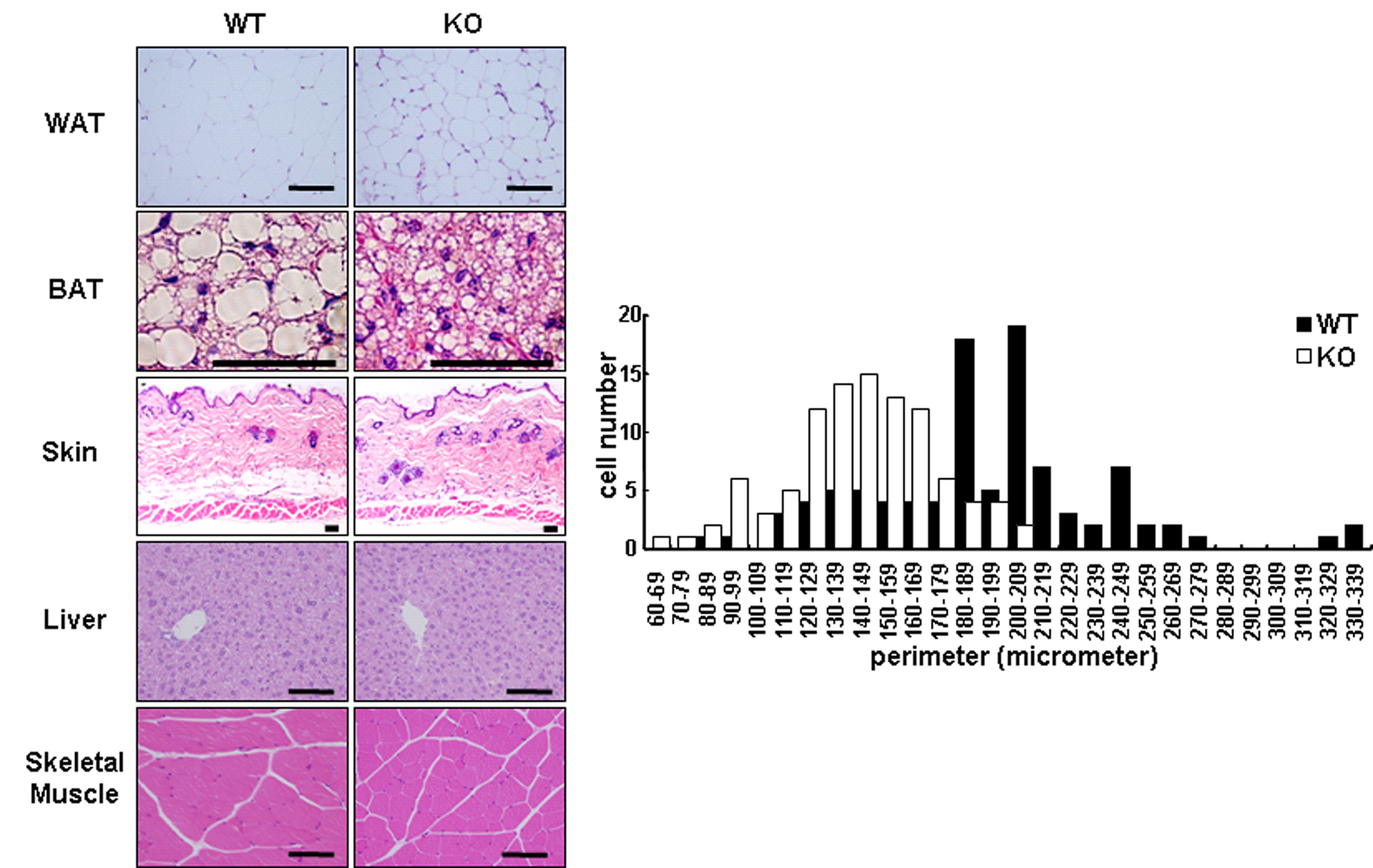

Supplement: Figure S2 — Histological analysis of epididymal WAT, BAT, skin, liver and skeletal muscle of 22 week-old male mice by hematoxylin and eosin staining. Scale bar, 50 µm. (Right graph) The measurement of cell perimeter of epididymal white adipose tissue (WAT) by Image J software. Representative adipocyte perimeter distribution of 200 cells from two animals per group (Median, 193 µm for WT; 144 µm for KO. P<0.001 by Mann-Whitney U test). (9.56 MB TIF) [file pone.0004240.s002.tif]

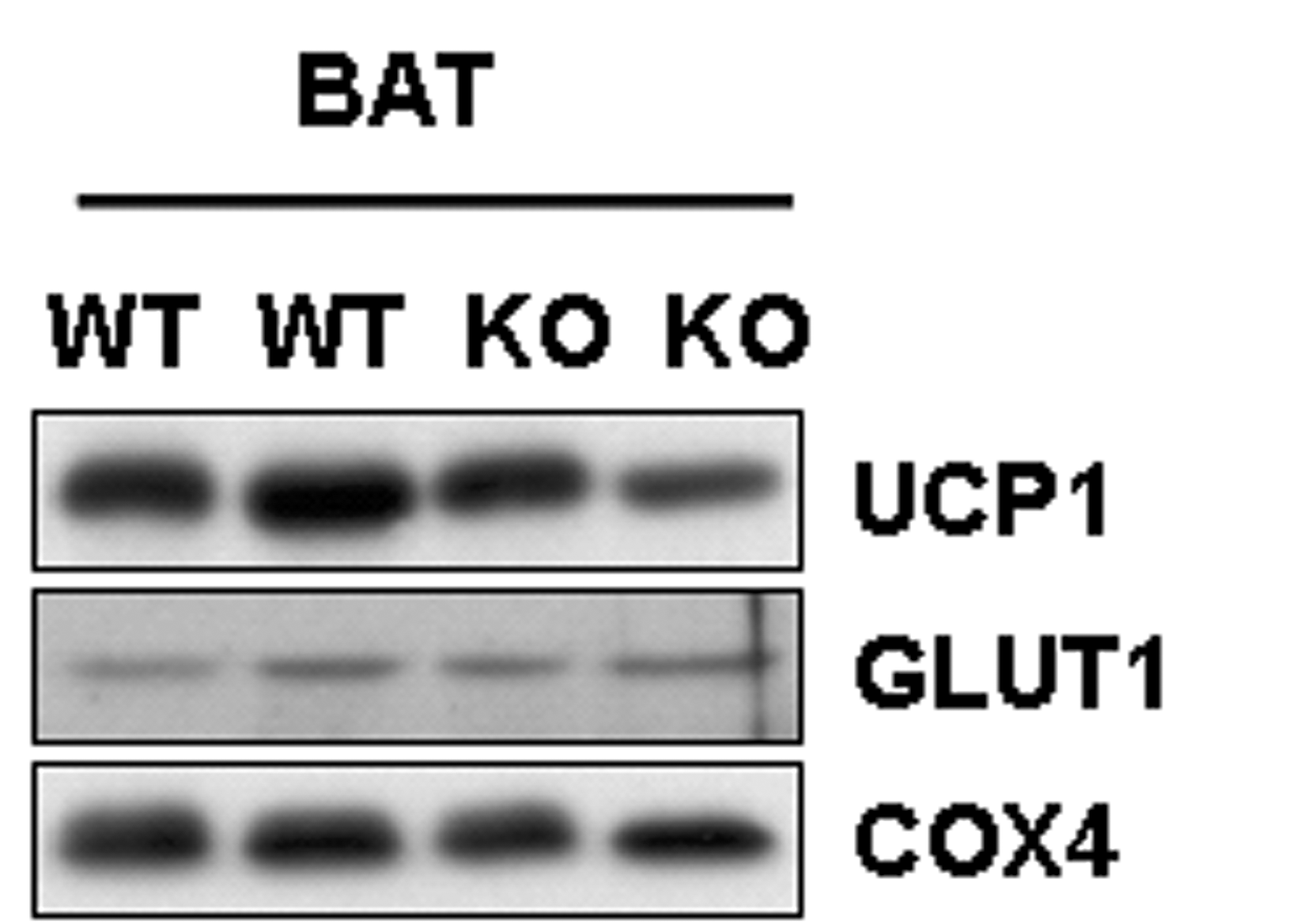

Supplement: Figure S3 — No difference in expression levels of UCP-1, GLUT-1 or COX-4 in brown adipose tissue between KRAP−/− and wild-type mice. Interscapular brown adipose tissue (BAT) was obtained from KRAP−/− (KO) and wild-type (WT) mice. Protein expression levels of UCP-1, GLUT-1 and COX-4 were determined by western blotting. UCP-1 Ab, GLUT-1 Ab and COX-4 Ab were purchased from Sigma (U6382), Santa Cruz (H-43) and Clontech (S2207), respectively. (1.94 MB TIF) [file pone.0004240.s003.tif]

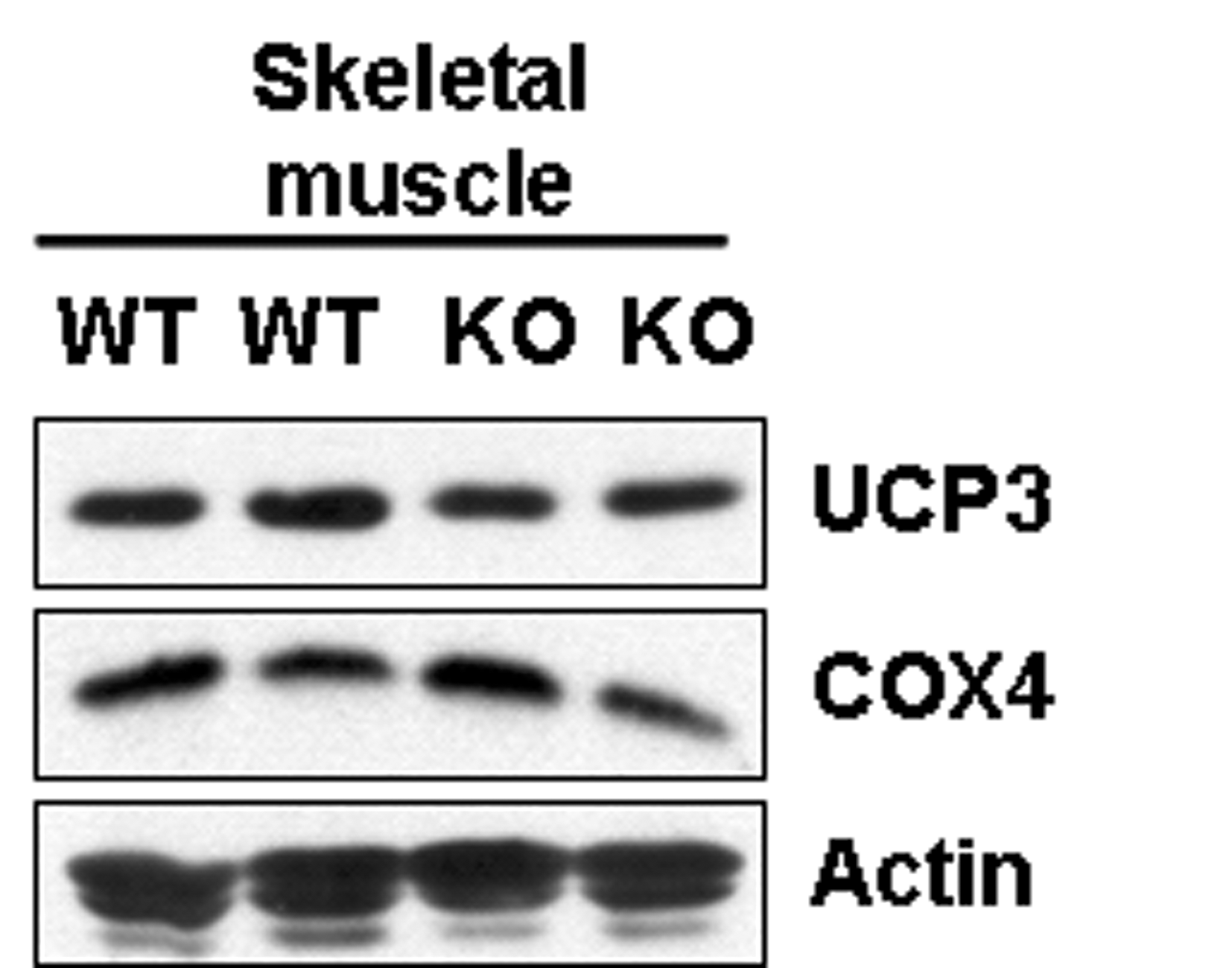

Supplement: Figure S4 — No difference in expression levels of UCP-3 or COX-4 in skeletal muscle between KRAP−/− and wild-type mice. Skeletal muscle was obtained from KRAP−/− (KO) and wild-type (WT) mice. Protein expression levels of UCP-3 and COX-4 were determined by western blotting. UCP-3 Ab, COX-4 Ab and Actin Ab were purchased from Sigma (U7757), Clontech (S2207) and Sigma (A2066), respectively. (2.15 MB TIF) [file pone.0004240.s004.tif]

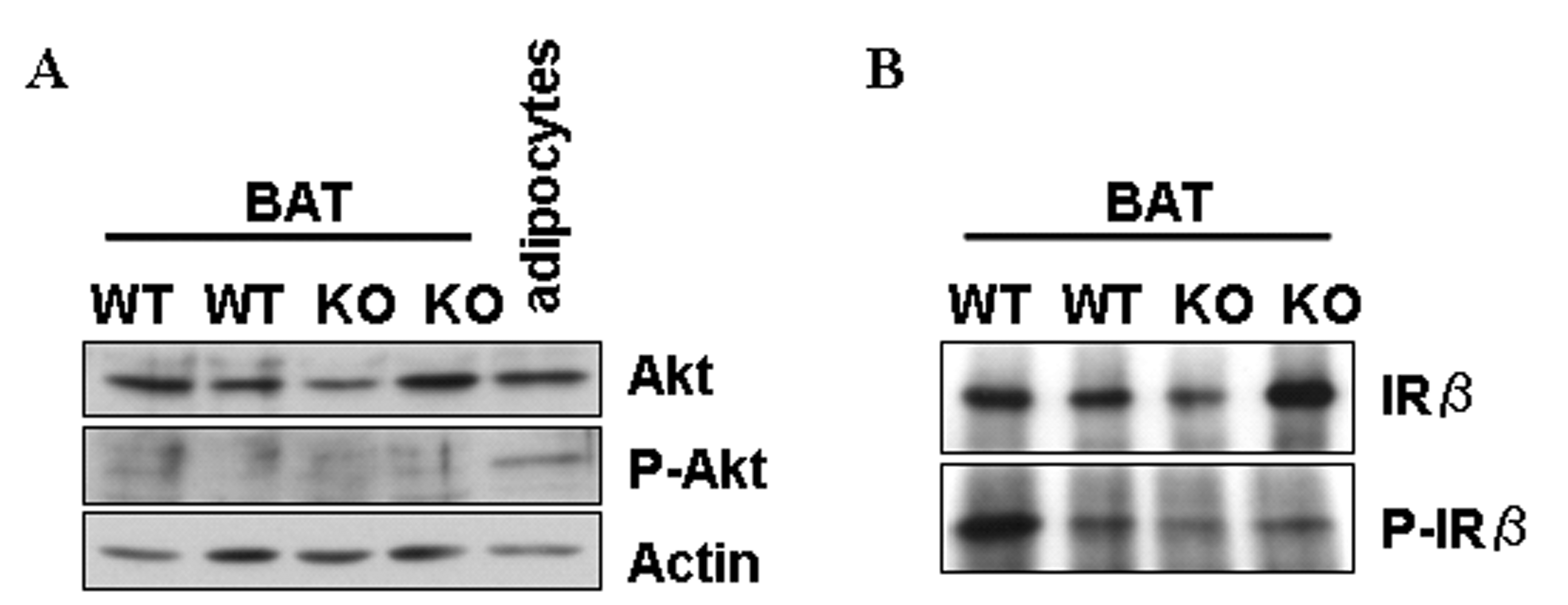

Supplement: Figure S5 — Absence of significant activations of Akt or Insulin receptor β in the KRAP−/−-BAT in fasted condition. Interscapular brown adipose tissue (BAT) was obtained from KRAP−/− (KO) and wild-type (WT) mice. (A) Protein expression levels of Akt, phospho-Akt (Ser473) (P-Akt) and Actin were determined by western blotting. As a positive control for P-Akt, lysate from cultured adipocytes treated with 100 nM of insulin for 5 min was used. Akt Ab, phospho-Akt (Ser473) Ab and Actin Ab were purchased from Cell Signaling (#9272), Cell Signaling (#9271) and Sigma (A2066), respectively. (B) Protein expression levels of total- and phospho-Insulin receptor (IR) β (Tyr972) were determined by western blotting. Immunoprecipitates with IRβ Ab were immunoblotted by IRβ Ab (C-19, Santa Cruz) or phospho-IRβ (Tyr972) Ab (44-800G, BIOSOURCE). (4.85 MB TIF) [file pone.0004240.s005.tif]

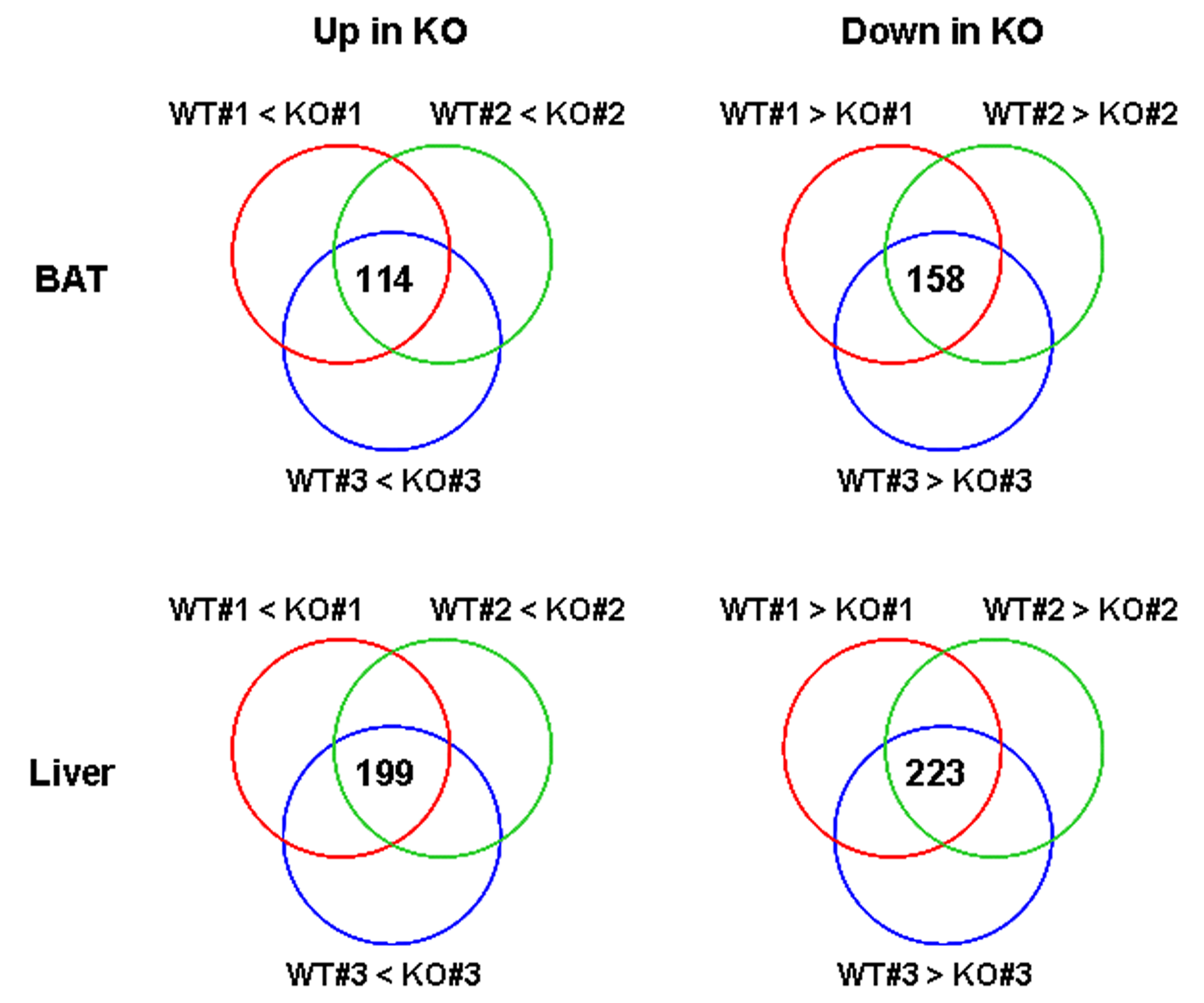

Supplement: Figure S6 — Upper Venn diagrams showed the up-regulated 114 probesets and down-regulated 158 probesets in KRAP−/−-brown adipose tissue. Lower Venn diagrams showed the up-regulated 119 probesets and down-regulated 223 probesets in KRAP−/−-liver. A cut-off value of 1.5-fold or more change between the KRAP−/− (KO) and the wild-type (WT) tissues was used. The data of the differentially expressed genes were provided in Table S3 and S4. (10.08 MB TIF) [file pone.0004240.s006.tif]
