# Supplementary material for: Altered Energy Homeostasis and Resistance to Diet-Induced Obesity in KRAP-Deficient Mice
Source: PLoS One. 2009 Jan 21;4(1):e4240. doi: 10.1371/journal.pone.0004240 (PMC2627767; doi:10.1371/journal.pone.0004240)
Supplement: Table S2 — Serum chemistry of wild-type and KRAP−/− mice. All samples were from mice on a standard diet and assessed for serum parameters as described in Methods. Data are presented as the mean±S.E.M.; *P<0.05 and P<0.01 compared with wild-type controls. n, number; FFA, free fatty acid; T3, triiodothyronine; T4, thyroxine. (0.05 MB DOC) [file pone.0004240.s008.doc]

**Standard diet**

Triglycerides

(mg/dl)

144 ± 14

103 ± 16

Cholesterol

(mg/dl)

76 ± 5

76 ± 6

FFA

(mEq/l)

0.46 ± 0.05

0.47 ± 0.08

**Wild-type**

***KRAP*^-/-^**

***n***

**Parameters (unit)**

10

10

10

Glucose

(mg/dl)

163 ± 7

9

Ketone

(mmol/l)

15

0.13 ± 0.02

0.14 ± 0.03

Insulin

(ng/ml)

1.42 ± 0.33

Leptin

(ng/ml)

3.77 ± 0.97

1.33 ± 0.18

Adiponectin

(μg/ml)

18 ± 1

T3

10

10

10

(ng/dl)

8

Glucagon

(pg/ml)

8

87 ± 5

329 ± 39

287 ± 45

73 ± 3*

0.60 ± 0.14*

24 ± 2

137 ± 4

Albumin

(g/dl)

10

2.70 ± 0.13

2.92 ± 0.08

IGF-1

(ng/ml)

8

615 ± 11

570 ± 16*

T4

(ng/ml)

10

28 ± 2

17 ± 2
,

Growth hormone

(ng/ml)

8

3.4 ± 0.7

3.2 ± 0.7
