# Supplementary material for: Altered Energy Homeostasis and Resistance to Diet-Induced Obesity in KRAP-Deficient Mice
Source: PLoS One. 2009 Jan 21;4(1):e4240. doi: 10.1371/journal.pone.0004240 (PMC2627767; doi:10.1371/journal.pone.0004240)
Supplement: Table S3 — Gene Ontology (GO) terms enriched in the down-regulated genes in KRAP−/−-BAT. Out of 158 down-regulated genes, 104 genes were found to have GO term annotations and subjected to GO term enrichment analysis. Expression data for 1.5-fold or more down- and up-regulated genes in KRAP−/−-BAT. Microarray gene expression analysis was performed on the brown adipose tissues from three pairs of KRAP−/− (KO) and the wild-type (WT) controls. (0.64 MB DOC) [file pone.0004240.s009.doc]

**Gene Ontology (GO) terms enriched in the down-regulated genes in *KRAP^-/-^***-BAT

| No. | Category | Genes  in  Category | % of  Genes in  Category | Genes  in List in  Category | % of  Genes in  List in  Category | p-Value |
| --- | --- | --- | --- | --- | --- | --- |
| 1 | GO:6633: fatty acid biosynthesis | 128 | 0.576 | 8 | 7.692 | 1.58E-07 |
| 2 | GO:16053: organic acid biosynthesis | 135 | 0.608 | 8 | 7.692 | 2.38E-07 |
| 3 | GO:46394: carboxylic acid biosynthesis | 135 | 0.608 | 8 | 7.692 | 2.38E-07 |
| 4 | GO:6631: fatty acid metabolism | 287 | 1.292 | 9 | 8.654 | 8.30E-06 |
| 5 | GO:44255: cellular lipid metabolism | 900 | 4.05 | 15 | 14.42 | 1.90E-05 |
| 6 | GO:42749: regulation of circadian sleep/wake cycle | 2 | 0.009 | 2 | 1.923 | 2.17E-05 |
| 7 | GO:45187: regulation of circadian sleep/wake cycle, sleep | 2 | 0.009 | 2 | 1.923 | 2.17E-05 |
| 8 | GO:50802: circadian sleep/wake cycle, sleep | 2 | 0.009 | 2 | 1.923 | 2.17E-05 |
| 9 | GO:8610: lipid biosynthesis | 445 | 2.003 | 10 | 9.615 | 4.57E-05 |
| 10 | GO:6629: lipid metabolism | 1055 | 4.748 | 15 | 14.42 | 0.000115 |
| 11 | GO:42745: circadian sleep/wake cycle | 4 | 0.018 | 2 | 1.923 | 0.000129 |
| 12 | GO:42752: regulation of circadian rhythm | 4 | 0.018 | 2 | 1.923 | 0.000129 |
| 13 | GO:6793: phosphorus metabolism | 1632 | 7.345 | 19 | 18.27 | 0.000186 |
| 14 | GO:6796: phosphate metabolism | 1632 | 7.345 | 19 | 18.27 | 0.000186 |
| 15 | GO:30431: sleep | 5 | 0.0225 | 2 | 1.923 | 0.000215 |
| 16 | GO:48512: circadian behavior | 6 | 0.027 | 2 | 1.923 | 0.000321 |
| 17 | GO:6801: superoxide metabolism | 29 | 0.131 | 3 | 2.885 | 0.000333 |
| 18 | GO:16310: phosphorylation | 1360 | 6.121 | 16 | 15.38 | 0.00057 |
| 19 | GO:30968: unfolded protein response | 35 | 0.158 | 3 | 2.885 | 0.000585 |
| 20 | GO:6468: protein amino acid phosphorylation | 1247 | 5.612 | 15 | 14.42 | 0.000686 |
| 21 | GO:45444: fat cell differentiation | 40 | 0.18 | 3 | 2.885 | 0.000868 |

**Down-regulated genes in *KRAP^-/-^***-BAT

| Gene Symbol | Description | Probe Set ID | Fold change KO#1vs.WT#1 | Fold change KO#2vs.WT#2 | Fold change KO#3vs.WT#3 | Genbank |
| --- | --- | --- | --- | --- | --- | --- |
| Abcd2 | ATP-binding cassette, sub-family D (ALD), member 2 | 1419748_at | 0.56 | 0.63 | 0.66 | NM_011994 |
| Acot11 | Acyl-CoA thioesterase 11 | 1429267_at | 0.44 | 0.27 | 0.64 | AW060409 |
| Adrb3 | Adrenergic receptor, beta 3 | 1421555_at | 0.40 | 0.51 | 0.27 | NM_013462 |
| Akr1c12 | Aldo-keto reductase family 1, member C12 | 1450455_s_at | 0.62 | 0.26 | 0.65 | AF177041 |
| Anxa2 | Annexin A2 | 1419091_a_at | 0.61 | 0.50 | 0.64 | NM_007585 |
| Atp4b | ATPase, H+/K+ exchanging, beta polypeptide | 1448911_at | 0.64 | 0.34 | 0.43 | NM_009724 |
| Braf | Braf transforming gene | 1445786_at | 0.62 | 0.41 | 0.39 | BB539247 |
| Camk2b | Calcium/calmodulin-dependent protein kinase II, beta | 1448676_at | 0.61 | 0.48 | 0.31 | NM_007595 |
| Casp12 | Caspase 12 | 1449297_at | 0.60 | 0.41 | 0.27 | NM_009808 |
| Ccdc3 | Coiled-coil domain containing 3 | 1428549_at | 0.58 | 0.26 | 0.55 | AK009833 |
| Ccdc25 | coiled-coil domain containing 25 | 1451799_at | 0.07 | 0.43 | 0.35 | BC025545 |
| Ccnd1 | Cyclin D1 | 1417419_at | 0.35 | 0.48 | 0.38 | NM_007631 |
| Ccnd1 | Cyclin D1 | 1417420_at | 0.52 | 0.54 | 0.46 | NM_007631 |
| Cdadc1 | Cytidine and dCMP deaminase domain containing 1 | 1458339_at | 0.37 | 0.39 | 0.61 | BB231078 |
| Cdkn2b | Cyclin-dependent kinase inhibitor 2B (p15, inhibits CDK4) | 1449152_at | 0.10 | 0.32 | 0.51 | AF059567 |
| Centa1 | Centaurin, alpha 1 | 1433556_at | 0.59 | 0.30 | 0.57 | AV264037 |
| Chac1 | ChaC, cation transport regulator-like 1 (E. coli) | 1451382_at | 0.60 | 0.51 | 0.58 | BC025169 |
| Cish | Cytokine inducible SH2-containing protein | 1448724_at | 0.47 | 0.64 | 0.40 | NM_009895 |
| Clec4b1 | C-type lectin domain family 4, member b1 | 1421685_at | 0.62 | 0.41 | 0.42 | NM_027218 |
| Clec7a | C-type lectin domain family 7, member a | 1420699_at | 0.42 | 0.66 | 0.51 | NM_020008 |
| Cops3 | COP9 (constitutive photomorphogenic) homolog, subunit 3 (Arabidopsis thaliana) | 1458259_x_at | 0.32 | 0.03 | 0.06 | BB283617 |
| Coro1c | Coronin, actin binding protein 1C | 1449660_s_at | 0.62 | 0.52 | 0.60 | AW548837 |
| Cxcl9 | Chemokine (C-X-C motif) ligand 9 | 1456907_at | 0.55 | 0.47 | 0.56 | BI104444 |
| Cycs | Cytochrome c, somatic | 1445484_at | 0.49 | 0.30 | 0.59 | BB741727 |
| Ddr1 | Discoidin domain receptor family, member 1 | 1456226_x_at | 0.55 | 0.37 | 0.64 | BB234940 |
| Dennd4a | Expressed sequence AI115600 | 1435975_at | 0.57 | 0.39 | 0.66 | BQ175496 |
| Depdc6 | DEP domain containing 6 | 1443579_s_at | 0.57 | 0.31 | 0.46 | AI957118 |
| Depdc6 | DEP domain containing 6 | 1428622_at | 0.58 | 0.34 | 0.52 | AK014624 |
| Dpysl4 | Dihydropyrimidinase-like 4 | 1418298_s_at | 0.23 | 0.48 | 0.02 | NM_011993 |
| Ela1 | Elastase 1, pancreatic | 1423693_at | 0.44 | 0.21 | 0.54 | BC011218 |
| Epb4.1l2 | Erythrocyte protein band 4.1-like 2 | 1433490_s_at | 0.53 | 0.59 | 0.66 | BE951907 |
| F8a | Factor 8-associated gene A | 1417412_at | 0.56 | 0.63 | 0.46 | NM_007978 |
| Fads3 | Fatty acid desaturase 3 | 1449219_at | 0.26 | 0.03 | 0.06 | BE652876 |
| Fads3 | Fatty acid desaturase 3 | 1435910_at | 0.41 | 0.21 | 0.28 | BM235658 |
| Fads3 | Fatty acid desaturase 3 | 1418773_at | 0.44 | 0.14 | 0.28 | BE652876 |
| Fgf1 | Fibroblast growth factor 1 | 1423136_at | 0.39 | 0.40 | 0.52 | AI649186 |
| Gcap14 | Granule cell antiserum positive 14 | 1452223_s_at | 0.45 | 0.52 | 0.61 | BM214039 |
| Ggta1 | Glycoprotein galactosyltransferase alpha 1, 3 | 1418483_a_at | 0.58 | 0.37 | 0.49 | AF297615 |
| Gimap9 | GTPase, IMAP family member 9 | 1437756_at | 0.34 | 0.61 | 0.14 | BF682515 |
| Glipr1 | GLI pathogenesis-related 1 (glioma) | 1424927_at | 0.66 | 0.48 | 0.55 | BC025083 |
| Gprk5 | G protein-coupled receptor kinase 5 | 1449514_at | 0.52 | 0.43 | 0.66 | BC019379 |
| Grb14 | Growth factor receptor bound protein 14 | 1417673_at | 0.43 | 0.46 | 0.47 | NM_016719 |
| Gss | Glutathione synthetase | 1446542_at | 0.63 | 0.45 | 0.42 | AW553564 |
| Hfe | Hemochromatosis | 1450702_at | 0.55 | 0.36 | 0.56 | AJ306425 |
| Hist1h1e | Histone cluster 1, H1e | 1445565_at | 0.41 | 0.63 | 0.63 | BE688907 |
| Hist2h3c2 | Histone cluster 2, H3c1 | 1422155_at | 0.28 | 0.41 | 0.59 | BC015270 |
| Hist3h2a | Histone cluster 3, H2a | 1455712_at | 0.41 | 0.62 | 0.44 | AI848909 |
| Hmgn3 | High mobility group nucleosomal binding domain 3 | 1431777_a_at | 0.45 | 0.41 | 0.51 | AK002970 |
| Hr | Hairless | 1435950_at | 0.49 | 0.37 | 0.32 | AV231755 |
| Hspb8 | Heat shock protein 8 | 1417013_at | 0.40 | 0.35 | 0.64 | AF250139 |
| Ifi203 | Interferon activated gene 203 | 1451567_a_at | 0.21 | 0.54 | 0.62 | BC008167 |
| Inhbb | Inhibin beta-B | 1426858_at | 0.66 | 0.47 | 0.61 | BB253137 |
| Jub | ajuba | 1421344_a_at | 0.63 | 0.30 | 0.48 | NM_010590 |
| Klhdc7a | Kelch domain containing 7A | 1456409_at | 0.41 | 0.48 | 0.63 | BB308961 |
| Ldlr | Low density lipoprotein receptor | 1421821_at | 0.52 | 0.52 | 0.44 | AF425607 |
| Lep | Leptin | 1422582_at | 0.31 | 0.24 | 0.18 | U18812 |
| Lysmd4 | LysM, putative peptidoglycan-binding, domain containing 4 | 1434093_at | 0.59 | 0.56 | 0.48 | AV376944 |
| Mal2 | Mal, T-cell differentiation protein 2 | 1427042_at | 0.46 | 0.59 | 0.59 | BB127697 |
| Map3k8 | Mitogen activated protein kinase kinase kinase 8 | 1419208_at | 0.61 | 0.63 | 0.64 | NM_007746 |
| Med18 | Mediator of RNA polymerase II transcription, subunit 18 homolog (yeast) | 1460462_at | 0.50 | 0.25 | 0.34 | AK012903 |
| Mmd | Monocyte to macrophage differentiation-associated | 1423488_at | 0.65 | 0.45 | 0.65 | BC021914 |
| Mmd | Monocyte to macrophage differentiation-associated | 1423489_at | 0.65 | 0.42 | 0.64 | BC021914 |
| Mpdz | Multiple PDZ domain protein | 1418664_at | 0.53 | 0.51 | 0.59 | AK019164 |
| Mpeg1 | Macrophage expressed gene 1 | 1427076_at | 0.53 | 0.58 | 0.56 | L20315 |
| Mrpl47 | Mitochondrial ribosomal protein L47 | 1443915_at | 0.63 | 0.54 | 0.57 | BE957160 |
| Nek6 | NIMA (never in mitosis gene a)-related expressed kinase 6 | 1425850_a_at | 0.50 | 0.18 | 0.49 | BC019524 |
| Nek6 | NIMA (never in mitosis gene a)-related expressed kinase 6 | 1423596_at | 0.51 | 0.17 | 0.45 | BB528391 |
| Npr3 | Natriuretic peptide receptor 3 | 1435184_at | 0.40 | 0.43 | 0.31 | BG066982 |
| Nrarp | Notch-regulated ankyrin repeat protein | 1417986_at | 0.62 | 0.61 | 0.40 | BI696369 |
| Nrip2 | Nuclear receptor interacting protein 2 | 1433792_at | 0.51 | 0.29 | 0.52 | AW491344 |
| Nrxn2 | Neurexin II | 1435908_at | 0.65 | 0.49 | 0.44 | BE949064 |
| Ntn1 | Netrin 1 | 1454974_at | 0.66 | 0.33 | 0.47 | BI143915 |
| P2rxl1 | Purinergic receptor P2X-like 1, orphan receptor | 1456925_at | 0.26 | 0.61 | 0.56 | AW490413 |
| Pamci | Peptidylglycine alpha-amidating monooxygenase COOH-terminal interactor | 1427942_at | 0.63 | 0.55 | 0.52 | BC028805 |
| Pcdh7 | Protocadherin 7 | 1437442_at | 0.41 | 0.49 | 0.27 | BG067986 |
| Pdzrn3 | PDZ domain containing RING finger 3 | 1416846_a_at | 0.64 | 0.66 | 0.55 | NM_018884 |
| Pex2 | Peroxin 2 | 1458932_at | 0.57 | 0.66 | 0.09 | BB639093 |
| Pgd | Phosphogluconate dehydrogenase | 1423706_a_at | 0.52 | 0.57 | 0.66 | BC014793 |
| Pik3r1 | Phosphatidylinositol 3-kinase, regulatory subunit, polypeptide 1 (p85 alpha) | 1425514_at | 0.38 | 0.33 | 0.58 | M60651 |
| Pkp2 | Plakophilin 2 | 1449799_s_at | 0.51 | 0.26 | 0.50 | AA516617 |
| Pkp2 | Plakophilin 2 | 1429183_at | 0.64 | 0.34 | 0.45 | AK005020 |
| Pnpla3 | Patatin-like phospholipase domain containing 3 | 1420655_at | 0.64 | 0.53 | 0.51 | NM_054088 |
| Ppm1a | Protein phosphatase 1A, magnesium dependent, alpha isoform | 1429500_at | 0.55 | 0.51 | 0.44 | C85630 |
| Ptgds | Prostaglandin D2 synthase (brain) | 1423860_at | 0.32 | 0.16 | 0.17 | AB006361 |
| Ptgds | Prostaglandin D2 synthase (brain) | 1423859_a_at | 0.33 | 0.18 | 0.18 | AB006361 |
| Pthr1 | Parathyroid hormone receptor 1 | 1417092_at | 0.66 | 0.32 | 0.65 | BC013446 |
| Ptplb | Protein tyrosine phosphatase-like (proline instead of catalytic arginine), member b | 1437329_at | 0.63 | 0.55 | 0.67 | BG067863 |
| Ptpn21 | Protein tyrosine phosphatase, non-receptor type 21 | 1453298_at | 0.49 | 0.17 | 0.61 | AK013777 |
| Rab6b | RAB6B, member RAS oncogene family | 1460617_s_at | 0.55 | 0.65 | 0.53 | AV220161 |
| Rbx1 | Ring-box 1 | 1416578_at | 0.62 | 0.35 | 0.56 | NM_019712 |
| Rer1 | RER1 retention in endoplasmic reticulum 1 homolog (S. cerevisiae) | 1460660_x_at | 0.66 | 0.54 | 0.57 | NM_026395 |
| Rock2 | Rho-associated coiled-coil containing protein kinase 2 | 1423592_at | 0.61 | 0.47 | 0.48 | BB761686 |
| S100b | S100 protein, beta polypeptide, neural | 1434342_at | 0.46 | 0.21 | 0.46 | BB316114 |
| S100b | S100 protein, beta polypeptide, neural | 1419383_at | 0.66 | 0.29 | 0.55 | NM_009115 |
| Sbk1 | SH3-binding kinase 1 | 1423978_at | 0.50 | 0.41 | 0.58 | BC025837 |
| Sbk1 | SH3-binding kinase 1 | 1451190_a_at | 0.59 | 0.42 | 0.63 | BC025837 |
| Sc5d | Sterol-C5-desaturase (fungal ERG3, delta-5-desaturase) homolog (S. cerevisae) | 1434520_at | 0.49 | 0.44 | 0.62 | AU067703 |
| Scd1 | Stearoyl-Coenzyme A desaturase 1 | 1415824_at | 0.12 | 0.66 | 0.64 | BG060909 |
| Scd1 | Stearoyl-Coenzyme A desaturase 1 | 1415823_at | 0.15 | 0.52 | 0.66 | BG060909 |
| Scd1 | Stearoyl-Coenzyme A desaturase 1 | 1415822_at | 0.16 | 0.41 | 0.67 | BG060909 |
| Serpina3c | Serine (or cysteine) peptidase inhibitor, clade A, member 3C | 1421564_at | 0.61 | 0.38 | 0.56 | NM_008458 |
| Sgcb | Sarcoglycan, beta (dystrophin-associated glycoprotein) | 1436678_at | 0.45 | 0.63 | 0.57 | AI844814 |
| Slc22a12 | Solute carrier family 22 (organic anion/cation transporter), member 12 | 1422898_s_at | 0.44 | 0.35 | 0.38 | NM_009203 |
| Slc2a5 | Solute carrier family 2 (facilitated glucose transporter), member 5 | 1416639_at | 0.22 | 0.23 | 0.49 | NM_019741 |
| Smc2 | Structural maintenance of chromosomes 2 | 1448635_at | 0.46 | 0.44 | 0.56 | NM_008017 |
| Smo | Smoothened homolog (Drosophila) | 1427048_at | 0.64 | 0.34 | 0.33 | AW555326 |
| Smyd4 | SET and MYND domain containing 4 | 1460053_at | 0.54 | 0.48 | 0.61 | BB021163 |
| Sncg | Synuclein, gamma | 1417788_at | 0.51 | 0.20 | 0.21 | NM_011430 |
| Sorl1 | Sortilin-related receptor, LDLR class A repeats-containing | 1453003_at | 0.39 | 0.50 | 0.65 | AK013519 |
| Sox9 | SRY-box containing gene 9 | 1451538_at | 0.22 | 0.46 | 0.57 | BC024958 |
| Spnb2 | Spectrin beta 2 | 1444089_at | 0.56 | 0.36 | 0.55 | AV016275 |
| Stxbp6 | Syntaxin binding protein 6 (amisyn) | 1425749_at | 0.44 | 0.18 | 0.25 | BC024598 |
| Sucnr1 | Succinate receptor 1 | 1418804_at | 0.60 | 0.40 | 0.54 | NM_032400 |
| Syn2 | Synapsin II | 1428460_at | 0.54 | 0.65 | 0.29 | AK013810 |
| Syn2 | Synapsin II | 1435511_at | 0.55 | 0.35 | 0.62 | BM898679 |
| Taok2 | TAO kinase 2 | 1438208_at | 0.54 | 0.50 | 0.42 | BM198170 |
| Tinag | Tubulointerstitial nephritis antigen | 1419314_at | 0.30 | 0.21 | 0.58 | BC010745 |
| Tlr5 | Toll-like receptor 5 | 1450242_at | 0.52 | 0.52 | 0.55 | NM_016928 |
| Tmem41b | Transmembrane protein 41B | 1437402_x_at | 0.46 | 0.58 | 0.50 | BB311030 |
| Tsga14 | Testis specific gene A14 | 1434576_at | 0.38 | 0.52 | 0.59 | BI648973 |
| Tssc1 | Tumor suppressing subtransferable candidate 1 | 1436955_at | 0.59 | 0.53 | 0.64 | BG065248 |
| Ugp2 | UDP-glucose pyrophosphorylase 2 | 1426460_a_at | 0.57 | 0.48 | 0.62 | AI788759 |
| Vav3 | Vav 3 oncogene | 1448600_s_at | 0.58 | 0.65 | 0.64 | BC027242 |
| Xrn2 | 5'-3' exoribonuclease 2 | 1422843_at | 0.55 | 0.49 | 0.66 | NM_011917 |
| Zfand3 | Zinc finger, AN1-type domain 3 | 1447850_x_at | 0.54 | 0.29 | 0.63 | BB076798 |
| Zfp81 | Zinc finger protein 81 | 1444076_at | 0.65 | 0.55 | 0.51 | BG063074 |
| Zfp84 | Zinc finger protein 84 | 1435916_at | 0.49 | 0.50 | 0.53 | BG067388 |
|  | RIKEN cDNA 0610010O12 gene | 1427878_at | 0.48 | 0.53 | 0.45 | AK002512 |
|  | RIKEN cDNA 1110008P14 gene | 1459890_s_at | 0.52 | 0.31 | 0.66 | C79326 |
|  | RIKEN cDNA 1110059M19 gene | 1429135_at | 0.26 | 0.16 | 0.16 | AV015858 |
|  | RIKEN cDNA 1500016O10 gene | 1452806_at | 0.38 | 0.53 | 0.63 | AK005271 |
|  | RIKEN cDNA 1500016O10 gene | 1452807_s_at | 0.41 | 0.28 | 0.62 | AK005271 |
|  | RIKEN cDNA 2610528K11 gene | 1428122_s_at | 0.65 | 0.65 | 0.59 | AK012178 |
|  | RIKEN cDNA 4732495E13 gene | 1427059_at | 0.57 | 0.63 | 0.53 | BB367207 |
|  | RIKEN cDNA 4930444A02 gene | 1424004_x_at | 0.47 | 0.64 | 0.62 | BC027296 |
|  | RIKEN cDNA 5730508B09 gene | 1429678_at | 0.55 | 0.60 | 0.61 | AK017758 |
|  | RIKEN cDNA 6720467C03 gene | 1451570_a_at | 0.66 | 0.36 | 0.63 | BC020162 |
|  | RIKEN cDNA A730020M07 gene | 1458484_at | 0.65 | 0.56 | 0.62 | AW319105 |
|  | Transcribed locus | 1441818_at | 0.17 | 0.34 | 0.64 | AI642706 |
|  | AV232649 RIKEN full-length enriched, 0 day neonate skin Mus musculus cDNA clone 4632416G03 3', mRNA sequence. | 1459270_at | 0.19 | 0.56 | 0.60 | AV232649 |
|  | RIKEN cDNA A530016L24 gene | 1438734_at | 0.32 | 0.66 | 0.59 | BB222714 |
|  | Sine oculis-related homeobox 4 homolog (Drosophila) | 1439753_x_at | 0.33 | 0.59 | 0.62 | AI893638 |
|  | BB481523 RIKEN full-length enriched, 13 days embryo lung Mus musculus cDNA clone D430004H12 3', mRNA sequence. | 1444430_at | 0.40 | 0.34 | 0.61 | BB481523 |
|  | Adult male urinary bladder cDNA, RIKEN full-length enriched library, clone:9530055M18 product:unclassifiable, full insert sequence | 1444531_at | 0.40 | 0.38 | 0.63 | AI847438 |
|  | 13 days embryo forelimb cDNA, RIKEN full-length enriched library, clone:5930438H13 product:unclassifiable, full insert sequence | 1455150_at | 0.44 | 0.66 | 0.51 | BB038506 |
|  | BB224790 RIKEN full-length enriched, adult male aorta and vein Mus musculus cDNA clone A530088E06 3', mRNA sequence. | 1455918_at | 0.45 | 0.47 | 0.39 | BB224790 |
|  | RIKEN cDNA A830093I24 gene | 1457617_at | 0.47 | 0.34 | 0.62 | AI851474 |
|  | AV166873 Mus musculus head C57BL/6J 13-day embryo Mus musculus cDNA clone 3110048N17, mRNA sequence. | 1438170_x_at | 0.51 | 0.48 | 0.66 | AV166873 |
|  | Transcribed locus, moderately similar to XP_982115.1 similar to gonadotropin inducible ovarian transcription factor 1 [Mus musculus] | 1443735_at | 0.55 | 0.46 | 0.47 | AV111366 |
|  | Transcribed locus | 1457011_at | 0.58 | 0.41 | 0.43 | BB797251 |
|  | RIKEN cDNA 4933413A10 gene | 1435796_at | 0.60 | 0.60 | 0.38 | AV280210 |
|  | CDNA sequence BC004044 | 1457648_x_at | 0.61 | 0.22 | 0.34 | BB084182 |
|  | Proteasome (prosome, macropain) 26S subunit, non-ATPase, 11 | 1440253_at | 0.62 | 0.62 | 0.57 | AV136581 |
|  | Adult male hippocampus cDNA, RIKEN full-length enriched library, clone:C630028A20 product:unclassifiable, full insert sequence | 1457215_at | 0.64 | 0.43 | 0.50 | BB429361 |
|  | Transcribed locus | 1440369_at | 0.64 | 0.40 | 0.61 | BE952603 |
|  | Transcribed locus | 1430245_at | 0.66 | 0.57 | 0.54 | BM209124 |
|  | RIKEN cDNA 2410066E13 gene | 1434581_at | 0.67 | 0.60 | 0.47 | BB167663 |
|  | AV218841 RIKEN full-length enriched, 12 days embryo head Mus musculus cDNA clone 3010073E09 3' similar to X83568 M.musculus mRNA for neuronatin-1, mRNA sequence. | 1423506_a_at | 0.67 | 0.29 | 0.17 | AV218841 |

**Up-regulated genes in *KRAP^-/-^***-BAT

| Gene Symbol | Description | Probe Set ID | Fold change KO#1vs.WT#1 | Fold change KO#2vs.WT#2 | Fold change KO#3vs.WT#3 | Genbank |
| --- | --- | --- | --- | --- | --- | --- |
| Acsm3 | Acyl-CoA synthetase medium-chain family member 3 | 1425559_a_at | 1.62 | 1.98 | 1.75 | AB022340 |
| Agpat6 | 1-acylglycerol-3-phosphate O-acyltransferase 6 (lysophosphatidic acid acyltransferase, zeta) | 1450776_at | 1.86 | 1.60 | 2.03 | NM_018743 |
| AI449310 | Expressed sequence AI449310 | 1455452_x_at | 1.88 | 1.52 | 2.70 | AW552116 |
| AI842396 | Expressed sequence AI842396 | 1448034_at | 1.79 | 1.62 | 1.87 | AA215276 |
| Alb1 | Albumin 1 | 1425260_at | 1.53 | 1.65 | 28.61 | BC024643 |
| AU067731 | Son cell proliferation protein | 1455634_at | 2.17 | 1.59 | 1.84 | AU067731 |
| Bcat2 | Branched chain aminotransferase 2, mitochondrial | 1425764_a_at | 2.09 | 2.29 | 2.62 | AF031467 |
| Bcl6 | B-cell leukemia/lymphoma 6 | 1450381_a_at | 3.24 | 2.48 | 1.68 | U41465 |
| Bicc1 | Bicaudal C homolog 1 (Drosophila) | 1423484_at | 2.11 | 1.65 | 1.52 | BM217996 |
| C1qtnf4 | C1q and tumor necrosis factor related protein 4 | 1417050_at | 2.50 | 6.39 | 1.99 | NM_026161 |
| Car14 | Carbonic anhydrase 14 | 1450725_s_at | 2.15 | 1.63 | 2.70 | NM_011797 |
| Cd163 | CD163 antigen | 1419144_at | 1.59 | 3.63 | 2.54 | NM_053094 |
| Cd209d | CD209d antigen | 1426183_a_at | 1.52 | 2.58 | 2.14 | AF440280 |
| Cdyl2 | Chromodomain protein, Y chromosome-like 2 | 1431139_at | 27.83 | 2.00 | 1.61 | BB634322 |
| Cfd | Complement factor D (adipsin) | 1417867_at | 1.82 | 1.52 | 1.78 | NM_013459 |
| Cfh | Complement component factor h | 1423153_x_at | 2.13 | 1.71 | 2.38 | AI987976 |
| Cfh | Complement component factor h | 1450876_at | 1.98 | 1.60 | 1.54 | AI987976 |
| Cirbp | Cold inducible RNA binding protein | 1416332_at | 1.62 | 2.25 | 1.62 | NM_007705 |
| Cldn1 | Claudin 1 | 1437932_a_at | 4.94 | 4.16 | 2.89 | AV227581 |
| Cldn1 | Claudin 1 | 1450014_at | 4.43 | 2.53 | 5.84 | NM_016674 |
| Col14a1 | Procollagen, type XIV, alpha 1 | 1427168_a_at | 2.19 | 2.49 | 1.55 | AJ131395 |
| Cyp2e1 | Cytochrome P450, family 2, subfamily e, polypeptide 1 | 1415994_at | 4.40 | 5.93 | 5.57 | NM_021282 |
| D1Ertd471e | DNA segment, Chr 1, ERATO Doi 471, expressed | 1436293_x_at | 1.51 | 1.65 | 1.68 | AI852300 |
| D9Wsu90e | DNA segment, Chr 9, Wayne State University 90, expressed | 1442409_at | 2.12 | 1.69 | 3.20 | BB166984 |
| Ddhd1 | DDHD domain containing 1 | 1427289_at | 2.30 | 3.91 | 1.85 | AV265534 |
| Ddx41 | DEAD (Asp-Glu-Ala-Asp) box polypeptide 41 | 1423814_at | 1.62 | 1.69 | 1.56 | BC011308 |
| Dio2 | Deiodinase, iodothyronine, type II | 1426081_a_at | 2.49 | 1.81 | 2.80 | AF177197 |
| Dnajc1 | DnaJ (Hsp40) homolog, subfamily C, member 1 | 1420501_at | 1.84 | 3.00 | 1.63 | NM_007869 |
| Dpyd | Dihydropyrimidine dehydrogenase | 1427945_at | 2.17 | 1.62 | 2.16 | BC028831 |
| Dpyd | Dihydropyrimidine dehydrogenase | 1427946_s_at | 1.72 | 1.59 | 1.80 | BC028831 |
| Dsc3 | Desmocollin 3 | 1434534_at | 2.09 | 2.12 | 2.29 | BB006344 |
| Entpd4 | Ectonucleoside triphosphate diphosphohydrolase 4 | 1438177_x_at | 1.98 | 1.72 | 1.72 | AV255351 |
| Entpd4 | Ectonucleoside triphosphate diphosphohydrolase 4 | 1447900_x_at | 1.66 | 2.03 | 1.53 | BB022415 |
| Errfi1 | ERBB receptor feedback inhibitor 1 | 1419816_s_at | 1.70 | 1.57 | 1.88 | AI788755 |
| Fcna | ficolin A | 1418243_at | 2.38 | 2.02 | 1.66 | NM_007995 |
| Flcn | Folliculin | 1438167_x_at | 1.89 | 1.53 | 1.53 | AV269574 |
| Gcnt2 | Glucosaminyl (N-acetyl) transferase 2, I-branching enzyme | 1430826_s_at | 3.16 | 1.64 | 2.64 | AK019924 |
| Gcnt2 | Mus musculus IGnT B mRNA for beta-1,6-N-acetylglucosaminyltransferase B, complete cds. | 1451733_at | 1.74 | 11.26 | 6.35 | AB037596 |
| Hnrpa3 | Heterogeneous nuclear ribonucleoprotein A3 | 1436261_at | 3.44 | 14.04 | 1.84 | BB723867 |
| Hpd | 4-hydroxyphenylpyruvic acid dioxygenase | 1424618_at | 2.79 | 2.12 | 2.68 | BC013343 |
| Hspb7 | Heat shock protein family, member 7 (cardiovascular) | 1421290_at | 2.07 | 2.62 | 1.55 | BG968304 |
| Il1rl2 | Interleukin 1 receptor-like 2 | 1434903_s_at | 1.74 | 2.91 | 1.57 | BG073776 |
| Impdh1 | Inosine 5'-phosphate dehydrogenase 1 | 1423239_at | 2.71 | 4.08 | 1.53 | BB351792 |
| Irf4 | Interferon regulatory factor 4 | 1421173_at | 3.06 | 2.65 | 5.02 | U34307 |
| Irf4 | Interferon regulatory factor 4 | 1421174_at | 1.83 | 3.41 | 1.91 | U34307 |
| Ivns1abp | Influenza virus NS1A binding protein | 1450084_s_at | 2.14 | 1.99 | 1.98 | NM_054102 |
| Ivns1abp | Influenza virus NS1A binding protein | 1425718_a_at | 1.68 | 2.45 | 1.94 | BC004092 |
| Kcnk3 | potassium channel, subfamily K, member 3 | 1426058_a_at | 1.97 | 4.60 | 2.00 | AF065162 |
| Klf15 | Kruppel-like factor 15 | 1448181_at | 2.30 | 3.18 | 1.69 | BC013486 |
| Lypla3 | Lysophospholipase 3 | 1422341_s_at | 1.76 | 3.25 | 1.71 | NM_133792 |
| Map4k5 | Mitogen-activated protein kinase kinase kinase kinase 5 | 1427376_a_at | 1.55 | 4.19 | 2.02 | BC002309 |
| Mgl1 | Macrophage galactose N-acetyl-galactosamine specific lectin 1 | 1419605_at | 2.01 | 1.52 | 1.67 | NM_010796 |
| Mmd2 | Monocyte to macrophage differentiation-associated 2 | 1438654_x_at | 3.14 | 2.57 | 2.32 | AV269411 |
| Mup1 | Major urinary protein 1 | 1420465_s_at | 4.47 | 2.44 | 37.71 | NM_031188 |
| Net1 | Neuroepithelial cell transforming gene 1 | 1421321_a_at | 1.88 | 3.34 | 1.74 | NM_019671 |
| Nsun6 | NOL1/NOP2/Sun domain family 6 | 1432012_a_at | 3.47 | 1.55 | 1.82 | AK016629 |
| Paip1 | Polyadenylate binding protein-interacting protein 1 | 1425521_at | 1.58 | 3.19 | 2.63 | BC019726 |
| Pcsk5 | Proprotein convertase subtilisin/kexin type 5 | 1437339_s_at | 3.16 | 1.86 | 2.18 | BB241731 |
| Plagl2 | Pleiomorphic adenoma gene-like 2 | 1417517_at | 1.57 | 1.50 | 1.50 | NM_018807 |
| Pon3 | paraoxonase 3; synonyms: AI786302, 2810004E20; Mus musculus paraoxonase 3 (Pon3), mRNA. | 1419298_at | 1.61 | 1.54 | 1.66 | NM_008897 |
| Ppm1k | Protein phosphatase 1K (PP2C domain containing) | 1452973_at | 1.66 | 1.73 | 2.43 | AK013741 |
| Rab17 | RAB17, member RAS oncogene family | 1422178_a_at | 4.12 | 9.95 | 2.41 | NM_008998 |
| Rbm35b | RNA binding motif protein 35b | 1433683_at | 7.52 | 1.79 | 2.74 | BF124648 |
| Retn | Resistin | 1449182_at | 2.72 | 1.55 | 1.70 | NM_022984 |
| Rgs2 | Regulator of G-protein signaling 2 | 1419247_at | 2.12 | 2.61 | 1.94 | AF215668 |
| Rnase4 | Ribonuclease, RNase A family 4 | 1438937_x_at | 1.59 | 2.03 | 2.45 | AI385586 |
| Rnf182 | Ring finger protein 182 | 1440086_at | 1.62 | 14.42 | 1.54 | BB428822 |
| Rod1 | ROD1 regulator of differentiation 1 (S. pombe) | 1424084_at | 1.59 | 1.57 | 2.31 | BB519382 |
| Rps6k | Ribosomal protein S6 kinase, polypeptide 1 | 1457562_at | 1.59 | 1.50 | 2.23 | BB195657 |
| Rrm2 | Ribonucleotide reductase M2 | 1448226_at | 2.04 | 1.84 | 1.51 | NM_009104 |
| Rwdd2 | RWD domain containing 2 | 1428963_at | 2.95 | 1.98 | 1.75 | AK006533 |
| Sbsn | Suprabasin | 1439630_x_at | 2.01 | 1.73 | 1.51 | AI844734 |
| Selenbp1 | Selenium binding protein 1 | 1450699_at | 1.94 | 1.54 | 1.90 | NM_009150 |
| Selenbp2 | Selenium binding protein 2 | 1417580_s_at | 1.67 | 1.70 | 1.76 | NM_019414 |
| Sfrs7 | Splicing factor, arginine/serine-rich 7 | 1436871_at | 1.75 | 1.79 | 1.64 | BE825013 |
| Sfxn2 | Sideroflexin 2 | 1435896_at | 1.55 | 2.18 | 1.60 | BG066686 |
| Sgk2 | Serum/glucocorticoid regulated kinase 2 | 1418739_at | 3.32 | 2.35 | 5.85 | NM_013731 |
| Slc27a2 | Solute carrier family 27 (fatty acid transporter), member 2 | 1416316_at | 1.53 | 1.53 | 2.19 | BC013442 |
| Slc4a4 | Solute carrier family 4 (anion exchanger), member 4 | 1434096_at | 2.16 | 1.52 | 2.42 | BB283443 |
| Slc5a6 | Solute carrier family 5 (sodium-dependent vitamin transporter), member 6 | 1435860_at | 1.58 | 1.64 | 1.59 | BF450030 |
| St3gal1 | ST3 beta-galactoside alpha-2,3-sialyltransferase 1 | 1418946_at | 2.39 | 3.80 | 1.56 | NM_009177 |
| Stat3 | Signal transducer and activator of transcription 3 | 1459961_a_at | 1.98 | 2.17 | 1.54 | BG069527 |
| Sytl1 | Synaptotagmin-like 1 | 1450240_a_at | 2.24 | 1.56 | 3.08 | NM_031393 |
| Tcn2 | Transcobalamin 2 | 1448200_at | 1.62 | 1.63 | 1.80 | NM_015749 |
| Tfrc | Transferrin receptor | 1422966_a_at | 4.66 | 3.00 | 1.54 | BB810450 |
| Thpo | Thrombopoietin | 1449569_at | 3.23 | 3.13 | 1.53 | NM_009379 |
| Timp2 | Tissue inhibitor of metalloproteinase 2 | 1420924_at | 1.75 | 1.53 | 2.02 | M93954 |
| Trf | Transferrin | 1425546_a_at | 1.67 | 1.85 | 1.70 | AF440692 |
| Wdr6 | WD repeat domain 6 | 1455940_x_at | 1.98 | 1.55 | 1.56 | BB453609 |
| Wdr61 | WD repeat domain 61 | 1434433_x_at | 2.72 | 1.62 | 1.70 | BF730671 |
|  | RIKEN cDNA 2310016C16 gene | 1424099_at | 1.80 | 1.63 | 2.08 | BC019664 |
|  | RIKEN cDNA 6230416J20 gene | 1429665_at | 1.77 | 2.05 | 1.60 | BB794620 |
|  | RIKEN cDNA 8430408G22 gene | 1433837_at | 2.80 | 2.21 | 1.82 | AV365503 |
|  | RIKEN cDNA C030002C11 gene | 1460033_at | 4.65 | 2.21 | 1.83 | BF469399 |
|  | RIKEN cDNA A930034L06 gene | 1434292_at | 2.73 | 2.34 | 1.57 | BI731047 |
|  | H3095A03-5 NIA Mouse 15K cDNA Clone Set Mus musculus cDNA clone H3095A03 5', mRNA sequence. | 1420310_at | 46.85 | 42.62 | 2.36 | BG083989 |
|  | RIKEN cDNA B230343A10 gene | 1442019_at | 8.75 | 6.16 | 1.65 | BB627097 |
|  | BB283832 RIKEN full-length enriched, adult retina Mus musculus cDNA clone A930103I05 3', mRNA sequence. | 1445562_at | 4.16 | 2.41 | 1.62 | BB283832 |
|  | RIKEN cDNA 1700019G17 gene | 1436670_x_at | 4.13 | 2.12 | 1.77 | BM214338 |
|  | BB096051 RIKEN full-length enriched, 12 days embryo, embryonic body between diaphragm region and neck Mus musculus cDNA clone 9430053N03 3', mRNA sequence. | 1459338_at | 3.53 | 15.62 | 1.65 | BB096051 |
|  | uw10b08.x1 Soares mouse 3NbMS Mus musculus cDNA clone IMAGE:3416247 3', mRNA sequence. | 1444017_at | 2.85 | 15.28 | 1.51 | BE692322 |
|  | AV243361 RIKEN full-length enriched, 0 day neonate head Mus musculus cDNA clone 4831419P14 3', mRNA sequence. | 1446719_at | 2.25 | 1.69 | 2.34 | AV243361 |
|  | Transcribed locus | 1457877_at | 2.14 | 4.01 | 3.94 | AW557111 |
|  | UI-M-BZ1-bky-d-04-0-UI.s1 NIH_BMAP_MHI2_S1 Mus musculus cDNA clone UI-M-BZ1-bky-d-04-0-UI 3', mRNA sequence. | 1459022_at | 2.14 | 1.88 | 1.53 | BF457812 |
|  | CDNA clone IMAGE:3493537 | 1437573_at | 2.07 | 1.61 | 2.08 | BF018351 |
|  | BB209878 RIKEN full-length enriched, 0 day neonate thymus Mus musculus cDNA clone A430094K05 3', mRNA sequence. | 1440773_at | 2.04 | 1.68 | 1.71 | BB209878 |
|  | Expressed sequence AU023762 | 1443888_at | 2.03 | 2.30 | 1.72 | BB426608 |
|  |  | 1449918_at | 2.01 | 4.30 | 2.19 | NM_027343 |
|  | H3034F11-3 NIA Mouse 15K cDNA Clone Set Mus musculus cDNA clone H3034F11 3', mRNA sequence. | 1444867_at | 1.67 | 1.63 | 1.90 | BG065741 |
|  | BB011550 RIKEN full-length enriched, 0 day neonate head Mus musculus cDNA clone 4831445M16 3' similar to AF127481 Homo sapiens non-ocogenic Rho GTPase-specific GTP exchange factor (proto-LBC), mRNA sequence. | 1443923_at | 1.59 | 1.52 | 2.04 | BB011550 |
|  | Transcribed locus | 1447830_s_at | 1.56 | 2.37 | 1.63 | BB034265 |
|  | Mus musculus 13 days embryo liver cDNA, RIKEN full-length enriched library, clone:2510001J03 product:Duffy blood group antigen, full insert sequence. | 1432273_a_at | 1.55 | 2.80 | 2.42 | AK010883 |
|  |  | AFFX-TransRecMur/X57349_3_at | 1.02 | 0.88 | 1.21 | AFFX-TransRecMur/X57349_3 |
|  |  | AFFX-TransRecMur/X57349_M_at | 0.98 | 0.83 | 1.20 | AFFX-TransRecMur/X57349_M |
