# Supplementary material for: Altered Energy Homeostasis and Resistance to Diet-Induced Obesity in KRAP-Deficient Mice
Source: PLoS One. 2009 Jan 21;4(1):e4240. doi: 10.1371/journal.pone.0004240 (PMC2627767; doi:10.1371/journal.pone.0004240)
Supplement: Table S4 — Gene Ontology (GO) terms enriched in the down-regulated genes in KRAP−/−-liver. Out of 223 down-regulated genes, 151 genes were found to have GO term annotations and subjected to GO term enrichment analysis. Expression data for 1.5-fold or more down- and up-regulated genes in KRAP−/−-liver. Microarray gene expression analysis was performed on the livers from three pairs of KRAP−/− (KO) and the wild-type (WT) controls. (0.95 MB DOC) [file pone.0004240.s010.doc]

**Gene Ontology (GO) terms enriched in the down-regulated genes in *KRAP^-/-^*-**liver

| No. | Category | Genes  in  Category | % of  Genes in  Category | Genes  in List in  Category | % of  Genes in  List in  Category | p-Value |
| --- | --- | --- | --- | --- | --- | --- |
| 1 | GO:16126: sterol biosynthesis | 56 | 0.252 | 25 | 16.560 | 3.72E-40 |
| 2 | GO:6694: steroid biosynthesis | 117 | 0.527 | 27 | 17.880 | 3.81E-34 |
| 3 | GO:6695: cholesterol biosynthesis | 47 | 0.212 | 21 | 13.910 | 7.64E-34 |
| 4 | GO:16125: sterol metabolism | 129 | 0.581 | 26 | 17.220 | 3.19E-31 |
| 5 | GO:8610: lipid biosynthesis | 445 | 2.003 | 37 | 24.500 | 9.99E-30 |
| 6 | GO:8202: steroid metabolism | 230 | 1.035 | 28 | 18.540 | 3.78E-27 |
| 7 | GO:8203: cholesterol metabolism | 120 | 0.540 | 22 | 14.570 | 1.51E-25 |
| 8 | GO:44255: cellular lipid metabolism | 900 | 4.050 | 40 | 26.490 | 7.08E-22 |
| 9 | GO:6066: alcohol metabolism | 445 | 2.003 | 30 | 19.870 | 1.84E-21 |
| 10 | GO:6629: lipid metabolism | 1055 | 4.748 | 40 | 26.490 | 2.11E-19 |
| 11 | GO:8299: isoprenoid biosynthesis | 31 | 0.140 | 10 | 6.623 | 6.10E-15 |
| 12 | GO:6720: isoprenoid metabolism | 46 | 0.207 | 10 | 6.623 | 5.14E-13 |
| 13 | GO:6084: acetyl-CoA metabolism | 82 | 0.369 | 11 | 7.285 | 9.14E-12 |
| 14 | GO:9058: biosynthesis | 2184 | 9.829 | 43 | 28.480 | 8.03E-11 |
| 15 | GO:15938: coenzyme A catabolism | 3 | 0.014 | 3 | 1.987 | 3.08E-07 |
| 16 | GO:9607: response to biotic stimulus | 1255 | 5.648 | 26 | 17.220 | 3.41E-07 |
| 17 | GO:6732: coenzyme metabolism | 297 | 1.337 | 12 | 7.947 | 9.35E-07 |
| 18 | GO:51186: cofactor metabolism | 354 | 1.593 | 13 | 8.609 | 9.49E-07 |
| 19 | GO:6700: C21-steroid hormone biosynthesis | 29 | 0.131 | 5 | 3.311 | 1.41E-06 |
| 20 | GO:6952: defense response | 1198 | 5.392 | 24 | 15.890 | 1.85E-06 |
| 21 | GO:6085: acetyl-CoA biosynthesis | 15 | 0.068 | 4 | 2.649 | 2.64E-06 |
| 22 | GO:8207: C21-steroid hormone metabolism | 34 | 0.153 | 5 | 3.311 | 3.22E-06 |

**Down-regulated genes in *KRAP^-/-^***-liver

| Gene Symbol | Description | Probe Set ID | Fold Change KO#1vs.WT#1 | Fold Change KO#2vs.WT#2 | Fold Change KO#3vs.WT#3 | Genbank |
| --- | --- | --- | --- | --- | --- | --- |
| Aacs | Acetoacetyl-CoA synthetase | 1423797_at | 0.14 | 0.25 | 0.16 | BC026817 |
| Aacs | Acetoacetyl-CoA synthetase | 1456081_a_at | 0.31 | 0.55 | 0.36 | AI987654 |
| Acacb | Acetyl-Coenzyme A carboxylase beta | 1427052_at | 0.24 | 0.21 | 0.38 | BC022940 |
| Acat2 | Acetyl-Coenzyme A acetyltransferase 2 | 1435630_s_at | 0.41 | 0.55 | 0.52 | AV148646 |
| Acly | ATP citrate lyase | 1425326_at | 0.31 | 0.47 | 0.49 | BI456232 |
| Acly | ATP citrate lyase | 1451666_at | 0.39 | 0.44 | 0.52 | BI456232 |
| Acsl3 | Acyl-CoA synthetase long-chain family member 3 | 1452771_s_at | 0.53 | 0.63 | 0.53 | AK012088 |
| Acss2 | Acyl-CoA synthetase short-chain family member 2 | 1422479_at | 0.20 | 0.33 | 0.33 | NM_019811 |
| Acss2 | Acyl-CoA synthetase short-chain family member 2 | 1422478_a_at | 0.23 | 0.38 | 0.41 | NM_019811 |
| Acvrl1 | Activin A receptor, type II-like 1 | 1451604_a_at | 0.50 | 0.39 | 0.65 | BC014291 |
| Adarb1 | Adenosine deaminase, RNA-specific, B1 | 1421480_a_at | 0.67 | 0.37 | 0.41 | NM_130895 |
| AI195470 | Expressed sequence AI195470 | 1440327_at | 0.61 | 0.62 | 0.66 | AA985897 |
| AI451617, | Expressed sequence AI451617 | 1435665_at | 0.43 | 0.29 | 0.61 | BM241342 |
| Anapc1 | Anaphase promoting complex subunit 1 | 1434443_at | 0.64 | 0.61 | 0.59 | AV113524 |
| Aqp8 | Aquaporin 8 | 1417828_at | 0.37 | 0.44 | 0.52 | NM_007474 |
| Arhgap26 | Rho GTPase activating protein 26 | 1444128_at | 0.17 | 0.28 | 0.33 | AI447325 |
| Art4 | ADP-ribosyltransferase 4 | 1449174_at | 0.05 | 0.56 | 0.58 | NM_026639 |
| B3galt1 | UDP-Gal:betaGlcNAc beta 1,3-galactosyltransferase, polypeptide 1 | 1441396_at | 0.58 | 0.40 | 0.20 | AV328619 |
| BC013672 | CDNA sequence BC013672 | 1451777_at | 0.63 | 0.53 | 0.53 | BC013672 |
| BC020489 | CDNA sequence BC020489 | 1424518_at | 0.36 | 0.49 | 0.59 | BC020489 |
| BC021614 | CDNA sequence BC021614 | 1424953_at | 0.48 | 0.48 | 0.63 | BC021614 |
| BC023105 | CDNA sequence BC023105 | 1425394_at | 0.66 | 0.34 | 0.51 | BC023105 |
| BC024137 | CDNA sequence BC024137 | 1427513_at | 0.31 | 0.45 | 0.36 | BI144810 |
| BC029214 | CDNA sequence BC029214 | 1442351_a_at | 0.32 | 0.38 | 0.49 | BB313029 |
| Ccdc25 | coiled-coil domain containing 25 | 1451799_at | 0.45 | 0.53 | 0.16 | BC025545 |
| Ccdc73 | Coiled-coil domain containing 73 | 1437189_x_at | 0.28 | 0.64 | 0.28 | BB311104 |
| Ccnc | Cyclin C | 1454144_a_at | 0.61 | 0.48 | 0.52 | AK014079 |
| Cd22 | CD22 antigen | 1419768_at | 0.62 | 0.44 | 0.65 | AF102134 |
| Chrna4 | Cholinergic receptor, nicotinic, alpha polypeptide 4 | 1456354_at | 0.39 | 0.37 | 0.08 | BB557207 |
| Clec4n | C-type lectin domain family 4, member n | 1425951_a_at | 0.60 | 0.52 | 0.64 | AF240358 |
| Cml2 | Camello-like 2 | 1421108_at | 0.53 | 0.62 | 0.59 | NM_053096 |
| Cxcl9 | Chemokine (C-X-C motif) ligand 9 | 1418652_at | 0.46 | 0.49 | 0.64 | NM_008599 |
| Cyb5b | Cytochrome b5 type B | 1448844_at | 0.47 | 0.54 | 0.55 | NM_025558 |
| Cyb5r3 | Cytochrome b5 reductase 3 | 1425329_a_at | 0.50 | 0.61 | 0.61 | AF332060 |
| Cyp17a1 | Cytochrome P450, family 17, subfamily a, polypeptide 1 | 1417017_at | 0.36 | 0.27 | 0.65 | NM_007809 |
| Cyp3a16 | Cytochrome P450, family 3, subfamily a, polypeptide 16 | 1421741_at | 0.27 | 0.01 | 0.00 | NM_007820 |
| Cyp3a41 | Cytochrome P450, family 3, subfamily a, polypeptide 41 | 1419704_at | 0.66 | 0.09 | 0.15 | NM_017396 |
| Cyp3a44 | Cytochrome P450, family 3, subfamily a, polypeptide 44 | 1426064_at | 0.28 | 0.17 | 0.16 | AB039380 |
| Cyp51 | Cytochrome P450, family 51 | 1450646_at | 0.32 | 0.54 | 0.25 | NM_020010 |
| Cyp51 | Cytochrome P450, family 51 | 1422533_at | 0.34 | 0.55 | 0.24 | NM_020010 |
| D11Lgp2e | DNA segment, Chr 11, Lothar Hennighausen 2, expressed | 1451426_at | 0.59 | 0.61 | 0.53 | AF316999 |
| D12Ertd647e | DNA segment, Chr 12, ERATO Doi 647, expressed | 1454757_s_at | 0.41 | 0.41 | 0.55 | AW554405 |
| D12Ertd647e | DNA segment, Chr 12, ERATO Doi 647, expressed | 1452956_a_at | 0.51 | 0.50 | 0.53 | BI655075 |
| Dct | Dopachrome tautomerase | 1418028_at | 0.42 | 0.30 | 0.66 | NM_010024 |
| Dhcr7 | 7-dehydrocholesterol reductase | 1448619_at | 0.25 | 0.42 | 0.22 | NM_007856 |
| Dll4 | Delta-like 4 (Drosophila) | 1421827_at | 0.56 | 0.53 | 0.66 | AK004739 |
| Dmrta1 | Doublesex and mab-3 related transcription factor like family A1 | 1441579_at | 0.66 | 0.54 | 0.37 | BB461344 |
| Echdc1 | Enoyl Coenzyme A hydratase domain containing 1 | 1419552_at | 0.54 | 0.63 | 0.65 | NM_025855 |
| Elovl1 | Elongation of very long chain fatty acids (FEN1/Elo2, SUR4/Elo3, yeast)-like 1 | 1425676_a_at | 0.57 | 0.66 | 0.57 | BC006735 |
| Elovl6 | ELOVL family member 6, elongation of long chain fatty acids (yeast) | 1417404_at | 0.35 | 0.50 | 0.65 | NM_130450 |
| Elovl6 | ELOVL family member 6, elongation of long chain fatty acids (yeast) | 1417403_at | 0.37 | 0.51 | 0.58 | NM_130450 |
| Erc2 | ELKS/RAB6-interacting/CAST family member 2 | 1434582_at | 0.22 | 0.51 | 0.64 | AV173869 |
| Fabp5 | Fatty acid binding protein 5, epidermal | 1416021_a_at | 0.64 | 0.43 | 0.29 | BC002008 |
| Fabp5 | Fatty acid binding protein 5, epidermal | 1416022_at | 0.66 | 0.38 | 0.29 | BC002008 |
| Fasn | Fatty acid synthase | 1423828_at | 0.28 | 0.41 | 0.40 | AF127033 |
| Fbxo39 | F-box protein 39 | 1443698_at | 0.59 | 0.51 | 0.52 | BB645745 |
| Fdft1 | Farnesyl diphosphate farnesyl transferase 1 | 1448130_at | 0.32 | 0.53 | 0.28 | NM_010191 |
| Fdft1 | Farnesyl diphosphate farnesyl transferase 1 | 1438322_x_at | 0.37 | 0.60 | 0.29 | BB028312 |
| Fdps | Farnesyl diphosphate synthetase | 1423418_at | 0.23 | 0.32 | 0.18 | BI247584 |
| Fmn1 | Formin 1 | 1439397_at | 0.56 | 0.46 | 0.46 | BB164513 |
| Fosl2 | Fos-like antigen 2 | 1422931_at | 0.41 | 0.53 | 0.65 | NM_008037 |
| Frmd4b | FERM domain containing 4B | 1426331_a_at | 0.62 | 0.65 | 0.54 | AF327857 |
| G1p2 | Mus musculus adult male hippocampus cDNA, RIKEN full-length enriched library, clone:2900034J12 product:interferon-stimulated protein (15 kDa), full insert sequence. | 1453939_x_at | 0.32 | 0.61 | 0.45 | AK019325 |
| G1p2 | Mus musculus adult male hippocampus cDNA, RIKEN full-length enriched library, clone:2900034J12 product:interferon-stimulated protein (15 kDa), full insert sequence. | 1431591_s_at | 0.49 | 0.53 | 0.39 | AK019325 |
| Gale | Galactose-4-epimerase, UDP | 1424140_at | 0.45 | 0.41 | 0.45 | BC027438 |
| Gbp2 | Guanylate nucleotide binding protein 2 | 1418240_at | 0.64 | 0.54 | 0.65 | NM_010260 |
| Gbp6 | Guanylate binding protein 6 | 1434380_at | 0.61 | 0.61 | 0.61 | BM241271 |
| Gna14 | Guanine nucleotide binding protein, alpha 14 | 1449848_at | 0.62 | 0.66 | 0.55 | NM_008137 |
| Golga3 | Golgi autoantigen, golgin subfamily a, 3 | 1419159_at | 0.65 | 0.46 | 0.54 | D78270 |
| Gvin1 | GTPase, very large interferon inducible 1 | 1429184_at | 0.50 | 0.39 | 0.59 | BM243571 |
| Hcls1 | Hematopoietic cell specific Lyn substrate 1 | 1418842_at | 0.45 | 0.62 | 0.37 | NM_008225 |
| Herc5 | Hect domain and RLD 5 | 1453757_at | 0.62 | 0.40 | 0.58 | AI639807 |
| Hes6 | Hairy and enhancer of split 6 (Drosophila) | 1436050_x_at | 0.37 | 0.52 | 0.54 | AI326893 |
| Hes6 | Hairy and enhancer of split 6 (Drosophila) | 1452021_a_at | 0.41 | 0.52 | 0.62 | AF247040 |
| Hmgcr | 3-hydroxy-3-methylglutaryl-Coenzyme A reductase | 1427229_at | 0.32 | 0.49 | 0.24 | BB123978 |
| Hmgcs1 | 3-hydroxy-3-methylglutaryl-Coenzyme A synthase 1 | 1433446_at | 0.31 | 0.59 | 0.29 | BB705380 |
| Hmgcs1 | 3-hydroxy-3-methylglutaryl-Coenzyme A synthase 1 | 1433443_a_at | 0.39 | 0.59 | 0.33 | BB705380 |
| Hmgcs1 | 3-hydroxy-3-methylglutaryl-Coenzyme A synthase 1 | 1433444_at | 0.43 | 0.63 | 0.40 | BB705380 |
| Hmgcs1 | 3-hydroxy-3-methylglutaryl-Coenzyme A synthase 1 | 1433445_x_at | 0.45 | 0.63 | 0.38 | BB705380 |
| Idi1 | Isopentenyl-diphosphate delta isomerase | 1423804_a_at | 0.28 | 0.52 | 0.31 | BC004801 |
| Idi1 | Isopentenyl-diphosphate delta isomerase | 1451122_at | 0.37 | 0.45 | 0.25 | BC004801 |
| Ifi44 | Interferon-induced protein 44 | 1423555_a_at | 0.40 | 0.57 | 0.48 | BB329808 |
| Ifi47 | Olfactory receptor 56 | 1417292_at | 0.51 | 0.60 | 0.60 | NM_008330 |
| Ifit1 | Interferon-induced protein with tetratricopeptide repeats 1 | 1450783_at | 0.43 | 0.47 | 0.37 | NM_008331 |
| Ifit3 | Interferon-induced protein with tetratricopeptide repeats 3 | 1449025_at | 0.40 | 0.39 | 0.45 | NM_010501 |
| Igh-6 | Immunoglobulin heavy chain 6 (heavy chain of IgM) | 1427351_s_at | 0.12 | 0.22 | 0.56 | BB226392 |
| Igh-6 | Immunoglobulin heavy chain 6 (heavy chain of IgM) | 1427329_a_at | 0.29 | 0.28 | 0.49 | AI326478 |
| Irf7 | Interferon regulatory factor 7 | 1417244_a_at | 0.46 | 0.54 | 0.53 | NM_016850 |
| Itgbl1 | Integrin, beta-like 1 | 1425039_at | 0.44 | 0.07 | 0.45 | BC020152 |
| Loxl4 | Lysyl oxidase-like 4 | 1421153_at | 0.17 | 0.43 | 0.49 | NM_053083 |
| Loxl4 | Lysyl oxidase-like 4 | 1450134_at | 0.42 | 0.36 | 0.44 | NM_053083 |
| Lss | Lanosterol synthase | 1420013_s_at | 0.22 | 0.37 | 0.17 | C77434 |
| Lss | Lanosterol synthase | 1426913_at | 0.33 | 0.44 | 0.28 | AK014742 |
| Ly6a | Lymphocyte antigen 6 complex, locus A | 1417185_at | 0.51 | 0.38 | 0.63 | BC002070 |
| Ly6e | Lymphocyte antigen 6 complex, locus E | 1453304_s_at | 0.46 | 0.43 | 0.59 | BM245572 |
| Mastl | Microtubule associated serine/threonine kinase-like | 1423525_at | 0.40 | 0.50 | 0.27 | BF021309 |
| Mid1ip1 | Mid1 interacting protein 1 (gastrulation specific G12-like (zebrafish)) | 1416840_at | 0.40 | 0.53 | 0.56 | NM_026524 |
| Mmab | Methylmalonic aciduria (cobalamin deficiency) type B homolog (human) | 1435097_at | 0.60 | 0.54 | 0.61 | BB772682 |
| Mobkl2a | MOB1, Mps One Binder kinase activator-like 2A (yeast) | 1434388_at | 0.61 | 0.43 | 0.56 | BB023868 |
| Mpa2l | Macrophage activation 2 like | 1447927_at | 0.39 | 0.34 | 0.37 | BG092512 |
| Mpa2l | Macrophage activation 2 like | 1438676_at | 0.56 | 0.31 | 0.41 | BM241485 |
| Mrpl19 | Mitochondrial ribosomal protein L19 | 1421913_at | 0.04 | 0.62 | 0.60 | BB041267 |
| Mtmr11 | Myotubularin related protein 11 | 1460550_at | 0.38 | 0.44 | 0.27 | BE952757 |
| Mtmr7 | BB431693 RIKEN full-length enriched, adult male hippocampus Mus musculus cDNA clone C630040D22 3', mRNA sequence. | 1447831_s_at | 0.38 | 0.55 | 0.49 | BB431693 |
| Mup1 | Major urinary protein 1 | 1434110_x_at | 0.54 | 0.38 | 0.58 | BF322785 |
| Mup1 | Major urinary protein 1 | 1430893_at | 0.09 | 0.29 | 0.41 | AK011413 |
| Mup3 | major urinary protein III; Mouse major urinary protein III (MUP III) mRNA, partial cds. | 1427631_x_at | 0.50 | 0.26 | 0.48 | M16359 |
| Mup5 | Major urinary protein 5 | 1426166_at | 0.52 | 0.55 | 0.65 | M16360 |
| Mvd | Mevalonate (diphospho) decarboxylase | 1448663_s_at | 0.14 | 0.35 | 0.15 | NM_138656 |
| Mvd | Mevalonate (diphospho) decarboxylase | 1417303_at | 0.20 | 0.23 | 0.17 | NM_138656 |
| Mvk | Mevalonate kinase | 1430619_a_at | 0.36 | 0.43 | 0.11 | AV324744 |
| Mvk | Mevalonate kinase | 1418052_at | 0.53 | 0.64 | 0.36 | BC005606 |
| Ndrg1 | N-myc downstream regulated gene 1 | 1450976_at | 0.46 | 0.48 | 0.40 | AI987929 |
| Ndrg1 | N-myc downstream regulated gene 1 | 1456174_x_at | 0.59 | 0.65 | 0.54 | AV309418 |
| Neu2 | Neuraminidase 2 | 1431936_a_at | 0.61 | 0.45 | 0.65 | AK009828 |
| Nfe2 | Nuclear factor, erythroid derived 2 | 1452001_at | 0.14 | 0.38 | 0.29 | L09600 |
| Nsdhl | NAD(P) dependent steroid dehydrogenase-like | 1416222_at | 0.39 | 0.47 | 0.29 | BC019945 |
| Nudt7 | nudix (nucleoside diphosphate linked moiety X)-type motif 7 | 1430896_s_at | 0.26 | 0.36 | 0.52 | AK008824 |
| Nudt7 | nudix (nucleoside diphosphate linked moiety X)-type motif 7 | 1453685_at | 0.32 | 0.16 | 0.58 | AK008824 |
| Nudt7 | Nudix (nucleoside diphosphate linked moiety X)-type motif 7 | 1431302_a_at | 0.33 | 0.45 | 0.57 | AK011172 |
| Oasl1 | 2'-5' oligoadenylate synthetase-like 1 | 1424339_at | 0.41 | 0.53 | 0.52 | AB067533 |
| Oasl2 | 2'-5' oligoadenylate synthetase-like 2 | 1453196_a_at | 0.43 | 0.59 | 0.50 | BQ033138 |
| Olfr480 | Olfactory receptor 480 | 1422351_at | 0.23 | 0.40 | 0.45 | NM_020291 |
| Paox | Polyamine oxidase (exo-N4-amino) | 1428859_at | 0.38 | 0.50 | 0.58 | AK010469 |
| Pclo | Piccolo (presynaptic cytomatrix protein) | 1419392_at | 0.32 | 0.36 | 0.32 | NM_011995 |
| Pcolce | Procollagen C-endopeptidase enhancer protein | 1448433_a_at | 0.60 | 0.50 | 0.65 | NM_008788 |
| Pcsk9 | AV010795 Mus musculus 18-day embryo C57BL/6J Mus musculus cDNA clone 1110029C19, mRNA sequence. | 1437453_s_at | 0.37 | 0.52 | 0.19 | AV010795 |
| Pdzk1ip1 | PDZK1 interacting protein 1 | 1455477_s_at | 0.61 | 0.37 | 0.55 | AA396586 |
| Pgd | Phosphogluconate dehydrogenase | 1423706_a_at | 0.52 | 0.60 | 0.63 | BC014793 |
| Pgk1 | Phosphoglycerate kinase 1 | 1439435_x_at | 0.53 | 0.23 | 0.53 | BB411302 |
| Pkib | Protein kinase inhibitor beta, cAMP dependent, testis specific | 1421137_a_at | 0.53 | 0.33 | 0.22 | AV047342 |
| Pltp | Phospholipid transfer protein | 1417963_at | 0.35 | 0.40 | 0.53 | NM_011125 |
| Pltp | Phospholipid transfer protein | 1456424_s_at | 0.51 | 0.55 | 0.60 | AI591480 |
| Pmvk | Phosphomevalonate kinase | 1427893_a_at | 0.27 | 0.48 | 0.27 | BI713896 |
| Ppard | Peroxisome proliferator activator receptor delta | 1439797_at | 0.36 | 0.33 | 0.28 | AW046066 |
| Psmb8 | Proteosome (prosome, macropain) subunit, beta type 8 (large multifunctional peptidase 7) | 1422962_a_at | 0.54 | 0.60 | 0.57 | NM_010724 |
| Psmb9 | Proteosome (prosome, macropain) subunit, beta type 9 (large multifunctional peptidase 2) | 1450696_at | 0.59 | 0.51 | 0.50 | NM_013585 |
| Pstpip2 | Proline-serine-threonine phosphatase-interacting protein 2 | 1455405_at | 0.33 | 0.41 | 0.42 | AV229693 |
| Pstpip2 | Proline-serine-threonine phosphatase-interacting protein 2 | 1421411_at | 0.37 | 0.37 | 0.49 | BC002123 |
| Pstpip2 | Proline-serine-threonine phosphatase-interacting protein 2 | 1421410_a_at | 0.41 | 0.39 | 0.42 | BC002123 |
| Rab14 | RAB14, member RAS oncogene family | 1419245_at | 0.59 | 0.55 | 0.51 | AV339290 |
| Rdh11 | Retinol dehydrogenase 11 | 1418760_at | 0.29 | 0.31 | 0.33 | AB030503 |
| Rdh11 | Retinol dehydrogenase 11 | 1449209_a_at | 0.31 | 0.31 | 0.28 | AB030503 |
| Rnd2 | Rho family GTPase 2 | 1422670_at | 0.62 | 0.54 | 0.59 | NM_009708 |
| Ropn1l | Ropporin 1-like | 1423959_at | 0.55 | 0.61 | 0.64 | AF305427 |
| Rsad2 | Radical S-adenosyl methionine domain containing 2 | 1436058_at | 0.62 | 0.62 | 0.57 | BB132493 |
| S3-12 | Plasma membrane associated protein, S3-12 | 1438913_x_at | 0.49 | 0.66 | 0.60 | AW322533 |
| Samd9l | Sterile alpha motif domain containing 9-like | 1460603_at | 0.50 | 0.64 | 0.59 | BB145092 |
| Sc4mol | Sterol-C4-methyl oxidase-like | 1423078_a_at | 0.29 | 0.48 | 0.23 | AK005441 |
| Sc5d | Sterol-C5-desaturase (fungal ERG3, delta-5-desaturase) homolog (S. cerevisae) | 1434520_at | 0.40 | 0.50 | 0.33 | AU067703 |
| Sc5d | Sterol-C5-desaturase (fungal ERG3, delta-5-desaturase) homolog (S. cerevisae) | 1451457_at | 0.49 | 0.58 | 0.47 | AB016248 |
| Sc5d | Sterol-C5-desaturase (fungal ERG3, delta-5-desaturase) homolog (S. cerevisae) | 1424709_at | 0.57 | 0.44 | 0.39 | AB016248 |
| Scd1 | Stearoyl-Coenzyme A desaturase 1 | 1415965_at | 0.22 | 0.47 | 0.46 | NM_009127 |
| Serpina12 | Serine (or cysteine) peptidase inhibitor, clade A (alpha-1 antiproteinase, antitrypsin), member 12 | 1421092_at | 0.14 | 0.20 | 0.21 | AK014346 |
| Serpina4-ps1 | Serine (or cysteine) peptidase inhibitor, clade A, member 4, pseudogene 1 | 1448092_x_at | 0.17 | 0.22 | 0.21 | AA267743 |
| Serpina4-ps1 | Serine (or cysteine) peptidase inhibitor, clade A, member 4, pseudogene 1 | 1444297_at | 0.38 | 0.26 | 0.12 | BF383739 |
| Slc13a2 | Solute carrier family 13 (sodium-dependent dicarboxylate transporter), member 2 | 1418857_at | 0.41 | 0.14 | 0.43 | BC013493 |
| Slc17a3 | Solute carrier family 17 (sodium phosphate), member 3 | 1418923_at | 0.60 | 0.66 | 0.50 | NM_134069 |
| Slc22a7 | Solute carrier family 22 (organic anion transporter), member 7 | 1451460_a_at | 0.29 | 0.35 | 0.44 | BC026598 |
| Slc43a1 | Solute carrier family 43, member 1 | 1453255_at | 0.62 | 0.53 | 0.62 | AK011417 |
| Slc45a4 | Solute carrier family 45, member 4 | 1441047_at | 0.39 | 0.40 | 0.22 | BB082429 |
| Slc6a9 | Solute carrier family 6 (neurotransmitter transporter, glycine), member 9 | 1431812_a_at | 0.55 | 0.55 | 0.45 | AK014572 |
| Slco1a1 | Solute carrier organic anion transporter family, member 1a1 | 1449844_at | 0.09 | 0.05 | 0.23 | AB031813 |
| Slco1a1 | Solute carrier organic anion transporter family, member 1a1 | 1420379_at | 0.11 | 0.05 | 0.39 | AB031813 |
| Spsb4 | SplA/ryanodine receptor domain and SOCS box containing 4 | 1451419_at | 0.28 | 0.65 | 0.62 | BC023083 |
| Sqle | Squalene epoxidase | 1415993_at | 0.24 | 0.44 | 0.16 | NM_009270 |
| Srd5a1 | Steroid 5 alpha-reductase 1 | 1454649_at | 0.37 | 0.19 | 0.62 | AV003635 |
| Stat1 | Signal transducer and activator of transcription 1 | 1450034_at | 0.47 | 0.66 | 0.47 | AW214029 |
| Stat1 | Signal transducer and activator of transcription 1 | 1450033_a_at | 0.66 | 0.63 | 0.46 | AW214029 |
| Stk38l | Serine/threonine kinase 38 like | 1435877_at | 0.53 | 0.57 | 0.48 | BB476811 |
| Stk38l | Serine/threonine kinase 38 like | 1435878_at | 0.60 | 0.62 | 0.51 | BB476811 |
| Sult5a1 | Sulfotransferase family 5A, member 1 | 1449816_at | 0.22 | 0.17 | 0.17 | NM_020564 |
| Suv420h2 | Suppressor of variegation 4-20 homolog 2 (Drosophila) | 1424059_at | 0.62 | 0.66 | 0.54 | BC024816 |
| Tgtp | T-cell specific GTPase | 1449009_at | 0.64 | 0.58 | 0.42 | NM_011579 |
| Thrsp | Thyroid hormone responsive SPOT14 homolog (Rattus) | 1422973_a_at | 0.18 | 0.30 | 0.57 | NM_009381 |
| Thrsp | Thyroid hormone responsive SPOT14 homolog (Rattus) | 1424737_at | 0.25 | 0.30 | 0.60 | BC009165 |
| Tiam2 | T-cell lymphoma invasion and metastasis 2 | 1423186_at | 0.31 | 0.28 | 0.52 | BM228957 |
| Tm7sf2 | RIKEN cDNA 1110014N23 gene | 1460684_at | 0.60 | 0.50 | 0.59 | BC014769 |
| Tm7sf3 | Transmembrane 7 superfamily member 3 | 1428098_a_at | 0.65 | 0.48 | 0.52 | AK010720 |
| Tmco6 | Transmembrane and coiled-coil domains 6 | 1451692_at | 0.64 | 0.37 | 0.53 | BC005637 |
| Tmem142c | Transmembrane protein 142C | 1434064_at | 0.62 | 0.61 | 0.61 | BQ175677 |
| Tmem14a | Transmembrane protein 14A | 1428447_at | 0.66 | 0.40 | 0.45 | AK017734 |
| Tnfsf10 | Tumor necrosis factor (ligand) superfamily, member 10 | 1459913_at | 0.50 | 0.53 | 0.24 | AI645293 |
| Trim12 | Tripartite motif protein 12 | 1437432_a_at | 0.42 | 0.43 | 0.50 | BM244351 |
| Ttll3 | Tubulin tyrosine ligase-like family, member 3 | 1436604_at | 0.63 | 0.60 | 0.39 | BG144520 |
| Uck2 | Uridine-cytidine kinase 2 | 1439740_s_at | 0.35 | 0.63 | 0.58 | AU018180 |
| Ugt2b5 | UDP glucuronosyltransferase 2 family, polypeptide B5 | 1423397_at | 0.55 | 0.29 | 0.63 | AI118428 |
| Urm1 | Ubiquitin related modifier 1 homolog (S. cerevisiae) | 1431752_a_at | 0.66 | 0.54 | 0.65 | AK012124 |
| Wt1 |  | 1425995_s_at | 0.26 | 0.40 | 0.21 | M55512 |
| Xrcc2 | X-ray repair complementing defective repair in Chinese hamster cells 2 | 1455335_at | 0.57 | 0.47 | 0.30 | AV338138 |
| Yap1 | Yes-associated protein 1 | 1416487_a_at | 0.67 | 0.54 | 0.53 | NM_009534 |
|  | RIKEN cDNA 1600029D21 gene | 1423933_a_at | 0.61 | 0.51 | 0.60 | BC022950 |
|  | RIKEN cDNA 1600029D21 gene | 1454254_s_at | 0.64 | 0.07 | 0.34 | AK002767 |
|  | RIKEN cDNA 1810008I18 gene | 1459141_at | 0.30 | 0.23 | 0.58 | BB667838 |
|  | RIKEN cDNA 1810022C23 gene | 1451588_at | 0.19 | 0.29 | 0.43 | BC014724 |
|  | RIKEN cDNA 2010001M09 gene | 1428947_at | 0.59 | 0.64 | 0.56 | AK008016 |
|  | RIKEN cDNA 2310041G17 gene | 1450881_s_at | 0.43 | 0.38 | 0.55 | AK010724 |
|  | RIKEN cDNA 2610019N06 gene | 1455769_at | 0.14 | 0.62 | 0.34 | AV221520 |
|  | RIKEN cDNA 2900009J20 gene | 1452989_at | 0.60 | 0.51 | 0.57 | BB315961 |
|  | RIKEN cDNA 2900024O10 gene | 1437704_at | 0.26 | 0.40 | 0.58 | AW494626 |
|  | RIKEN cDNA 3110045C21 gene | 1430401_at | 0.39 | 0.25 | 0.25 | AK014177 |
|  | RIKEN cDNA 4933403J19 gene | 1432748_at | 0.26 | 0.48 | 0.57 | AK016636 |
|  | RIKEN cDNA 5830462P14 gene | 1432997_at | 0.59 | 0.54 | 0.56 | AK018031 |
|  | RIKEN cDNA 9030607L17 gene | 1452809_at | 0.66 | 0.35 | 0.13 | AK018541 |
|  | RIKEN cDNA 9030619P08 gene | 1443889_at | 0.61 | 0.54 | 0.64 | AI789751 |
|  | RIKEN cDNA 9130221J18 gene | 1446368_at | 0.22 | 0.09 | 0.24 | AV377066 |
|  | RIKEN cDNA 9330112F22 gene | 1458972_at | 0.55 | 0.56 | 0.28 | BM250288 |
|  | RIKEN cDNA A530040E14 gene | 1456493_at | 0.24 | 0.53 | 0.56 | BG072319 |
|  | BB541054 RIKEN full-length enriched, 0 day neonate eyeball Mus musculus cDNA clone E130111P19 3', mRNA sequence. | 1443138_at | 0.23 | 0.22 | 0.30 | BB541054 |
|  | C76313 Mouse 3.5-dpc blastocyst cDNA Mus musculus cDNA clone J0008E09 3' similar to M.musculus gene for dodecenoyl-CoA delta-isomerase, exon 3, mRNA, mRNA sequence. | 1443312_at | 0.28 | 0.12 | 0.36 | C76313 |
|  | CDNA, clone:Y2G0135G14, strand:plus, reference:ENSEMBL:Mouse-Transcript-ENST:ENSMUST00000022462, based on BLAT search | 1447816_x_at | 0.29 | 0.63 | 0.62 | BB059395 |
|  | Predicted gene, EG624219 | 1427422_at | 0.34 | 0.44 | 0.62 | BM122014 |
|  | mr29d03.x1 Soares mouse 3NbMS Mus musculus cDNA clone IMAGE:598853 3', mRNA sequence. | 1449608_a_at | 0.35 | 0.57 | 0.46 | AI447620 |
|  | H3145A04-3 NIA Mouse 15K cDNA Clone Set Mus musculus cDNA clone H3145A04 3', mRNA sequence. | 1459168_at | 0.36 | 0.48 | 0.17 | BG075241 |
|  | BB471557 RIKEN full-length enriched, 12 days embryo eyeball Mus musculus cDNA clone D230039L18 3', mRNA sequence. | 1439169_at | 0.38 | 0.62 | 0.55 | BB471557 |
|  | Transcribed locus | 1446834_at | 0.40 | 0.37 | 0.50 | AV367948 |
|  | H3103G03-3 NIA Mouse 15K cDNA Clone Set Mus musculus cDNA clone H3103G03 3', mRNA sequence. | 1458872_at | 0.45 | 0.08 | 0.26 | BG071837 |
|  | K0834F04-3 NIA Mouse 8.5-dpc Whole Embryo cDNA Library (Long) Mus musculus cDNA clone NIA:K0834F04 IMAGE:30081663 3', mRNA sequence. | 1447615_at | 0.48 | 0.35 | 0.34 | BM249512 |
|  | id79f01.x1 Melton Normalized Mixed Mouse Pancreas 1 N1-MMS1 Mus musculus cDNA clone IMAGE:5669497 3', mRNA sequence. | 1444207_at | 0.56 | 0.60 | 0.19 | BM070003 |
|  | AV328153 RIKEN full-length enriched, adult male medulla oblongata Mus musculus cDNA clone 6330439H12 3', mRNA sequence. | 1459817_at | 0.56 | 0.25 | 0.63 | AV328153 |
|  | vm83b02.x1 Knowles Solter mouse blastocyst B1 Mus musculus cDNA clone IMAGE:1004811 3', mRNA sequence. | 1458713_at | 0.58 | 0.61 | 0.63 | AI503056 |
|  | Adult male thymus cDNA, RIKEN full-length enriched library, clone:5830448K13 product:unclassifiable, full insert sequence | 1457746_at | 0.64 | 0.53 | 0.22 | BG801845 |
|  | BB199132 RIKEN full-length enriched, 0 day neonate thymus Mus musculus cDNA clone A430012C18 3', mRNA sequence. | 1441576_at | 0.65 | 0.59 | 0.51 | BB199132 |

**Up-regulated genes in *KRAP^-/-^***-liver

| Gene Symbol | Description | Probe Set ID | Fold Change KO#1vs.WT#1 | Fold Change KO#2vs.WT#2 | Fold Change KO#3vs.WT#3 | Genbank |
| --- | --- | --- | --- | --- | --- | --- |
| Abca6 | ATP-binding cassette, sub-family A (ABC1), member 6 | 1453817_at | 2.75 | 2.06 | 2.10 | AK018242 |
| Acaa1a | Acetyl-Coenzyme A acyltransferase 1A | 1456011_x_at | 1.50 | 1.55 | 1.76 | BB210491 |
| Aco1 | BB758846 RIKEN full-length enriched, melanocyte Mus musculus cDNA clone G270103J12 3', mRNA sequence. | 1447080_at | 2.04 | 1.80 | 1.59 | BB758846 |
| AI426953 | Expressed sequence AI426953 | 1442018_at | 9.81 | 2.17 | 2.61 | BE632903 |
| Akap13 | BB011550 RIKEN full-length enriched, 0 day neonate head Mus musculus cDNA clone 4831445M16 3' similar to AF127481 Homo sapiens non-ocogenic Rho GTPase-specific GTP exchange factor (proto-LBC), mRNA sequence. | 1443923_at | 2.28 | 3.17 | 1.79 | BB011550 |
| Alg14 | Asparagine-linked glycosylation 14 homolog (yeast) | 1419116_at | 2.04 | 13.56 | 2.03 | AA198774 |
| Arl4a | ADP-ribosylation factor-like 4A | 1425411_at | 3.36 | 2.51 | 4.05 | BC003725 |
| Arrdc4 | Sterol O-acyltransferase 1 | 1424759_at | 2.13 | 1.51 | 1.95 | BC017528 |
| Atp2b1 | ATPase, Ca++ transporting, plasma membrane 1 | 1428936_at | 1.95 | 2.00 | 2.03 | BI080417 |
| Atrx | Alpha thalassemia/mental retardation syndrome X-linked homolog (human) | 1420946_at | 1.80 | 2.01 | 1.81 | BB825830 |
| B230206F22Rik | RIKEN cDNA B230206F22 gene | 1438838_at | 1.75 | 1.75 | 2.19 | BG141977 |
| Baz2b | Bromodomain adjacent to zinc finger domain, 2B | 1442971_at | 1.78 | 5.63 | 3.02 | BG069765 |
| BC017647 | CDNA sequence BC017647 | 1424663_at | 1.57 | 1.62 | 1.95 | BC017647 |
| Bhlhb9 | Basic helix-loop-helix domain containing, class B9 | 1428512_at | 2.90 | 1.89 | 1.56 | AK012577 |
| Braf | BB141769 RIKEN full-length enriched, adult female vagina Mus musculus cDNA clone 9930012E13 3', mRNA sequence. | 1442749_at | 1.52 | 1.77 | 3.36 | BB141769 |
| C77805 | expressed sequence C77805 | 1445490_at | 1.72 | 2.24 | 2.10 | C77805 |
| Cacng5 | Calcium channel, voltage-dependent, gamma subunit 5 | 1426330_at | 1.61 | 3.22 | 1.68 | AF458900 |
| Cbl | Casitas B-lineage lymphoma | 1455886_at | 13.25 | 1.86 | 2.45 | BB385581 |
| Ccar1 | Cell division cycle and apoptosis regulator 1 | 1436157_at | 1.96 | 1.74 | 1.65 | AW538049 |
| Cdc2l5 | Cell division cycle 2-like 5 (cholinesterase-related cell division controller) | 1440833_at | 2.11 | 1.78 | 3.37 | BG070538 |
| Cep57 | Centrosomal protein 57 | 1452983_at | 1.51 | 1.85 | 1.55 | AW457682 |
| Cln8 | Ceroid-lipofuscinosis, neuronal 8 | 1455745_at | 1.66 | 1.71 | 2.10 | BM249363 |
| Cpox | Coproporphyrinogen oxidase | 1440747_at | 3.19 | 1.99 | 2.88 | BB745944 |
| Cpox | Coproporphyrinogen oxidase | 1422492_at | 2.04 | 1.94 | 1.52 | BG067254 |
| Cpsf6 | Cleavage and polyadenylation specific factor 6 | 1437372_at | 2.99 | 1.93 | 1.92 | BB335087 |
| Crebbp | CREB binding protein | 1436983_at | 3.35 | 1.92 | 1.76 | BG069466 |
| Cwf19l2 | CWF19-like 2, cell cycle control (S. pombe) | 1453688_at | 2.56 | 1.53 | 1.73 | AK014327 |
| Cyb561d1 | Cytochrome b-561 domain containing 1 | 1459837_at | 1.58 | 2.28 | 2.86 | BB016212 |
| D17Ertd165e | DNA segment, Chr 17, ERATO Doi 165, expressed | 1446118_at | 2.63 | 1.70 | 1.93 | BG066166 |
| D1Ertd471e | DNA segment, Chr 1, ERATO Doi 471, expressed | 1436894_at | 1.60 | 2.52 | 1.85 | BB324138 |
| D1Ertd471e | DNA segment, Chr 1, ERATO Doi 471, expressed | 1436221_at | 1.56 | 2.67 | 2.24 | BG067625 |
| Dag1 | Dystroglycan 1 | 1426779_x_at | 1.98 | 2.00 | 1.52 | BG094386 |
| Ddx6 | DEAD (Asp-Glu-Ala-Asp) box polypeptide 6 | 1439122_at | 3.32 | 1.66 | 1.87 | BF226295 |
| Ddx6 | DEAD (Asp-Glu-Ala-Asp) box polypeptide 6 | 1424598_at | 2.78 | 2.62 | 1.80 | BC021452 |
| Defb1 | Defensin beta 1 | 1419491_at | 1.55 | 1.79 | 1.83 | BC024380 |
| Defb1 | Defensin beta 1 | 1419492_s_at | 1.51 | 1.86 | 1.91 | BC024380 |
| Dicer1 | Dicer1, Dcr-1 homolog (Drosophila) | 1427941_at | 2.15 | 1.63 | 1.55 | AF430845 |
| Dnajb14 | DnaJ (Hsp40) homolog, subfamily B, member 14 | 1430561_at | 2.31 | 1.93 | 1.68 | BE952491 |
| Dnajc1 | DnaJ (Hsp40) homolog, subfamily C, member 1 | 1459791_at | 3.90 | 4.64 | 4.26 | AV129500 |
| E030016H06Rik | RIKEN cDNA E030016H06 gene | 1440443_at | 1.51 | 1.74 | 1.51 | BB531351 |
| Epb4.1 | Erythrocyte protein band 4.1 | 1444150_at | 3.13 | 2.55 | 2.10 | BB462549 |
| Evi5 | Ecotropic viral integration site 5 | 1417513_at | 2.25 | 1.64 | 1.69 | AI255184 |
| Gabarapl2 | Gamma-aminobutyric acid (GABA-A) receptor-associated protein-like 2 | 1441376_at | 1.81 | 1.87 | 1.56 | BB426412 |
| Gapvd1 | GTPase activating protein and VPS9 domains 1 | 1444066_at | 2.23 | 1.71 | 2.32 | BB176698 |
| Glra1 | Glycine receptor, alpha 1 subunit | 1422277_at | 1.59 | 2.33 | 1.73 | NM_020492 |
| Glrx | Glutaredoxin | 1416593_at | 1.67 | 1.64 | 1.67 | AF276917 |
| Gmps | Guanine monphosphate synthetase | 1455057_at | 3.72 | 1.65 | 1.59 | AV308908 |
| Gna13 | Guanine nucleotide binding protein, alpha 13 | 1430295_at | 1.81 | 1.69 | 1.53 | BG094302 |
| Gsk3b | Glycogen synthase kinase 3 beta | 1439949_at | 2.13 | 1.50 | 1.93 | BG063622 |
| Hdac2 | Histone deacetylase 2 | 1439704_at | 2.99 | 2.22 | 1.52 | BB461532 |
| Hgd | Homogentisate 1, 2-dioxygenase | 1458049_at | 2.12 | 1.73 | 1.68 | BB667582 |
| Hmmr | hyaluronan mediated motility receptor (RHAMM) | 1429871_at | 1.54 | 1.86 | 1.98 | AK020144 |
| Hook3 | Hook homolog 3 (Drosophila) | 1443857_at | 2.75 | 1.56 | 1.87 | BB825115 |
| Hook3 | Hook homolog 3 (Drosophila) | 1439196_at | 1.54 | 1.61 | 1.61 | BB476531 |
| Iapls3-12 | Plasma membrane associated protein, S3-12 | 1418595_at | 3.90 | 3.30 | 1.78 | NM_020568 |
| Igh-6 | Immunoglobulin heavy chain 6 (heavy chain of IgM) | 1427756_x_at | 54.33 | 2.30 | 1.98 | M60430 |
| Ing3 | Inhibitor of growth family, member 3 | 1422806_x_at | 2.53 | 2.56 | 1.84 | BB020556 |
| Inhbb | Inhibin beta-B | 1426858_at | 3.73 | 1.83 | 2.28 | BB253137 |
| Itga4 | Integrin alpha 4 | 1456498_at | 45.76 | 1.88 | 2.11 | BB284583 |
| Itih5 | AV239969 RIKEN full-length enriched, 10 day neonate skin Mus musculus cDNA clone 4732437H13 3', mRNA sequence. | 1441946_at | 1.77 | 1.92 | 1.70 | AV239969 |
| Itsn2 | Intersectin 2 | 1423184_at | 2.60 | 2.41 | 1.91 | AI326108 |
| Kif1b | Kinesin family member 1B | 1451642_at | 2.29 | 1.81 | 1.77 | BE199508 |
| Lepr | leptin receptor | 1425644_at | 2.06 | 2.99 | 1.61 | U42467 |
| Lpin2 | Lipin 2 | 1446316_at | 4.34 | 2.88 | 2.11 | BB378708 |
| Luc7l2 | LUC7-like 2 (S. cerevisiae) | 1445717_at | 9.92 | 2.49 | 1.95 | BB283604 |
| Mapre1 | Microtubule-associated protein, RP/EB family, member 1 | 1428820_at | 3.18 | 1.68 | 2.29 | BB464192 |
| March7 | Membrane-associated ring finger (C3HC4) 7 | 1440966_at | 3.11 | 2.20 | 1.70 | BB248730 |
| Mllt4 | Myeloid/lymphoid or mixed lineage-leukemia translocation to 4 homolog (Drosophila) | 1439073_at | 1.65 | 1.67 | 1.92 | BB821719 |
| Mtss1 | Metastasis suppressor 1 | 1446284_at | 1.80 | 2.15 | 2.07 | BB157298 |
| Muc6 | mucin 6, gastric | 1441297_at | 5.18 | 1.51 | 2.09 | BG147188 |
| Myo6 | myosin VI | 1421120_at | 1.99 | 1.80 | 1.72 | NM_008662 |
| Mysm1 | Myb-like, SWIRM and MPN domains 1 | 1460159_at | 1.64 | 3.03 | 1.73 | BB372805 |
| Ncor1 | Nuclear receptor co-repressor 1 | 1423201_at | 2.09 | 1.68 | 1.58 | U22016 |
| Nfe2l2 | Nuclear factor, erythroid derived 2, like 2 | 1457117_at | 3.92 | 1.57 | 3.30 | AV248273 |
| Nipbl | Nipped-B homolog (Drosophila) | 1430309_at | 4.51 | 2.09 | 1.74 | BC025455 |
| Nisch | Nischarin | 1430151_at | 2.09 | 1.92 | 1.94 | AK014314 |
| Orm2 | Orosomucoid 2 | 1420438_at | 1.80 | 5.73 | 2.00 | NM_011016 |
| Pan3 | PAN3 polyA specific ribonuclease subunit homolog (S. cerevisiae) | 1437883_s_at | 2.60 | 1.53 | 2.03 | AV305314 |
| Pde6c | Phosphodiesterase 6C, cGMP specific, cone, alpha prime | 1450830_a_at | 2.97 | 2.99 | 2.41 | NM_033614 |
| Pigt |  | AFFX-18SRNAMur/X00686_5_at | 1.60 | 3.29 | 1.73 | AFFX-18SRNAMur/X00686_5 |
| Plekha1 | Pleckstrin homology domain containing, family A (phosphoinositide binding specific) member 1 | 1457491_at | 2.40 | 9.67 | 1.68 | BM238642 |
| Postn | Periostin, osteoblast specific factor | 1423606_at | 1.72 | 1.58 | 1.85 | BI110565 |
| Prpf38b | PRP38 pre-mRNA processing factor 38 (yeast) domain containing B | 1456506_at | 1.82 | 2.53 | 2.67 | BB479992 |
| Psip1 | PC4 and SFRS1 interacting protein 1 | 1457731_at | 1.75 | 1.97 | 1.74 | BB247595 |
| Rad23b | RAD23b homolog (S. cerevisiae) | 1456822_at | 2.02 | 1.55 | 1.54 | BB482313 |
| Ramp2 | Receptor (calcitonin) activity modifying protein 2 | 1418188_a_at | 2.20 | 2.84 | 1.68 | AF146523 |
| Rbm3 | RBM3; Mus musculus RNA-binding motif protein 3 mRNA, complete cds. | 1422660_at | 2.02 | 2.29 | 2.18 | AY052560 |
| [Rbm45](http://www.informatics.jax.org/javawi2/servlet/WIFetch?page=markerDetail&key=81803) | Developmentally regulated RNA binding protein 1 | 1437904_at | 4.94 | 5.64 | 5.49 | BB821609 |
| Rgs16 | Regulator of G-protein signaling 16 | 1451452_a_at | 3.13 | 4.78 | 2.22 | U72881 |
| Rgs16 | Regulator of G-protein signaling 16 | 1426037_a_at | 2.44 | 12.51 | 4.36 | U94828 |
| Rnf170 | Ring finger protein 170 | 1437207_at | 1.81 | 2.58 | 1.63 | BF458266 |
| Rreb1 | Ras responsive element binding protein 1 | 1434741_at | 2.25 | 1.69 | 1.52 | BB306052 |
| Rrm2b | Ribonucleotide reductase M2 B (TP53 inducible) | 1437221_at | 10.02 | 3.27 | 4.22 | BB702377 |
| Rufy3 | RUN and FYVE domain containing 3 | 1442786_s_at | 6.45 | 3.42 | 14.86 | BB461022 |
| Rufy3 | RUN and FYVE domain containing 3 | 1424402_at | 4.88 | 2.68 | 5.11 | AW494299 |
| Sae1 | SUMO1 activating enzyme subunit 1 | 1440185_x_at | 1.87 | 1.97 | 1.98 | BB293365 |
| Saps3 | SAPS domain family, member 3 | 1454030_at | 2.05 | 1.51 | 2.98 | AK018652 |
| Setx | Senataxin | 1440459_at | 2.57 | 1.59 | 1.73 | BB767941 |
| Slc4a4 | Solute carrier family 4 (anion exchanger), member 4 | 1421225_a_at | 2.23 | 1.57 | 1.60 | NM_018760 |
| Slc6a8 | Solute carrier family 6 (neurotransmitter transporter, creatine), member 8 | 1417116_at | 1.99 | 1.52 | 1.86 | BG069516 |
| Sltm | SAFB-like, transcription modulator | 1429624_at | 1.64 | 1.95 | 1.68 | BG068009 |
| Spag5 | Sperm associated antigen 5 | 1441988_at | 1.97 | 1.91 | 2.22 | AI482429 |
| Srrm2 | Serine/arginine repetitive matrix 2 | 1437638_at | 2.57 | 1.56 | 1.96 | BB821417 |
| St3gal5 | ST3 beta-galactoside alpha-2,3-sialyltransferase 5 | 1460241_a_at | 2.19 | 1.56 | 1.61 | BB829192 |
| Thoc2 | THO complex 2 | 1438736_at | 1.73 | 1.63 | 1.93 | BB703762 |
| Thumpd3 | THUMP domain containing 3 | 1454061_at | 2.50 | 4.11 | 1.68 | AK017246 |
| Tia1 | cytotoxic granule-associated RNA binding protein 1 | 1431708_a_at | 1.64 | 1.77 | 1.86 | AK009502 |
| Tloc1 | Translocation protein 1 | 1444811_at | 3.16 | 2.89 | 2.31 | BM293801 |
| Tmem56 | Transmembrane protein 56 | 1456718_at | 1.58 | 2.12 | 1.57 | BB768133 |
| Tnfaip8 | Tumor necrosis factor, alpha-induced protein 8 | 1442753_at | 4.19 | 3.55 | 1.59 | BF321807 |
| Tpr | Translocated promoter region | 1456112_at | 2.81 | 1.76 | 1.59 | AW554765 |
| Trim24 | Tripartite motif protein 24 | 1446295_at | 1.85 | 2.61 | 4.42 | BB325847 |
| Trio | Triple functional domain (PTPRF interacting) | 1457492_at | 2.74 | 2.82 | 1.74 | AW553130 |
| Trove2 | TROVE domain family, member 2 | 1436533_at | 2.76 | 1.58 | 2.45 | BQ176653 |
| Usp27x | Ubiquitin specific peptidase 27, X chromosome | 1436004_at | 2.15 | 1.86 | 2.84 | BB021271 |
| Usp36 | Ubiquitin specific peptidase 36 | 1456094_at | 2.96 | 2.96 | 2.11 | BG063007 |
| Wdr42a | WD repeat domain 42A | 1453662_at | 1.92 | 1.55 | 2.90 | AK020987 |
| Wnk1 | WNK lysine deficient protein kinase 1 | 1436746_at | 1.83 | 1.94 | 1.62 | BI692255 |
| Xdh | K0208G08-3 NIA Mouse Unfertilized Egg cDNA Library (Long) Mus musculus cDNA clone NIA:K0208G08 IMAGE:30045487 3', mRNA sequence. | 1424609_a_at | 1.70 | 1.60 | 1.85 | BM225255 |
| Xist | Inactive X specific transcripts | 1427262_at | 1.93 | 1.65 | 1.60 | L04961 |
| Xpr1 | Xenotropic and polytropic retrovirus receptor 1 | 1437958_at | 2.03 | 2.62 | 2.09 | BG066856 |
| Zc3h11a | Zinc finger CCCH type containing 11A | 1455998_at | 2.45 | 1.59 | 1.69 | BM232738 |
| Zfp207 | Zinc finger protein 207 | 1438515_at | 3.06 | 1.91 | 1.53 | AI646720 |
| Zp3 | Zona pellucida glycoprotein 3 | 1419007_at | 1.77 | 1.58 | 1.75 | NM_011776 |
|  | RIKEN cDNA 1110003F05 gene | 1429735_at | 1.58 | 1.65 | 1.73 | BB659256 |
|  | RIKEN cDNA 1810055G02 gene | 1425921_a_at | 2.80 | 1.79 | 1.59 | BC019471 |
|  | RIKEN cDNA 2610024E20 gene | 1442897_at | 2.23 | 1.70 | 1.64 | BB556313 |
|  | RIKEN cDNA 2810474O19 gene | 1437110_at | 3.11 | 2.04 | 2.94 | BM232998 |
|  | RIKEN cDNA 2900019G14 gene | 1439940_at | 2.75 | 2.10 | 1.84 | AV328280 |
|  | RIKEN cDNA 3300001P08 gene | 1451485_at | 1.61 | 1.66 | 1.66 | AW536179 |
|  | RIKEN cDNA 4930535B03 gene | 1434817_s_at | 3.12 | 4.37 | 1.53 | BM206427 |
|  | RIKEN cDNA 4933412E12 gene | 1447711_x_at | 2.25 | 1.62 | 3.08 | BB265147 |
|  | RIKEN cDNA 5830409B07 gene | 1429927_at | 1.75 | 2.90 | 2.58 | AK017913 |
|  | RIKEN cDNA 6430510M02 gene | 1457218_at | 3.85 | 2.55 | 1.51 | BB296225 |
|  | RIKEN cDNA 6820431F20 gene | 1429882_at | 2.90 | 2.05 | 1.74 | AK012880 |
|  | RIKEN cDNA 9130002K18 gene | 1431525_at | 1.71 | 1.54 | 2.56 | AK018586 |
|  | RIKEN cDNA 9130404D08 gene | 1451993_at | 1.98 | 2.47 | 1.66 | BC023401 |
|  | RIKEN cDNA 9430010O03 gene | 1433598_at | 2.38 | 1.72 | 1.54 | BG093966 |
|  | RIKEN cDNA 9430013L17 gene | 1433241_at | 1.69 | 1.77 | 1.83 | AK020414 |
|  | RIKEN cDNA A930004J17 gene | 1457760_at | 1.97 | 1.90 | 1.68 | BB243507 |
|  | EST AA407452 | 1456791_at | 7.45 | 1.95 | 1.65 | BG063017 |
|  | BB469078 RIKEN full-length enriched, 12 days embryo eyeball Mus musculus cDNA clone D230022F05 3', mRNA sequence. | 1457064_at | 15.63 | 1.53 | 1.79 | BB469078 |
|  | Predicted gene, EG620313 | 1434588_x_at | 7.46 | 2.34 | 2.29 | AI181686 |
|  | C0845B10-3 NIA Mouse Blastocyst cDNA Library (Long) Mus musculus cDNA clone NIA:C0845B10 IMAGE:30029301 3', mRNA sequence. | 1457483_at | 6.76 | 1.65 | 2.18 | BM214036 |
|  | BB168460 RIKEN full-length enriched, 16 days neonate thymus Mus musculus cDNA clone A130098O11 3', mRNA sequence. | 1446700_at | 6.46 | 2.85 | 2.56 | BB168460 |
|  | H3153A11-3 NIA Mouse 15K cDNA Clone Set Mus musculus cDNA clone H3153A11 3', mRNA sequence. | 1440396_at | 5.25 | 2.26 | 2.54 | BG075893 |
|  | H3034G07-3 NIA Mouse 15K cDNA Clone Set Mus musculus cDNA clone H3034G07 3', mRNA sequence. | 1442531_at | 4.25 | 5.21 | 1.51 | BG065748 |
|  | L0545H06-3 NIA Mouse Newborn Heart cDNA Library Mus musculus cDNA clone L0545H06 3', mRNA sequence. | 1446950_at | 4.11 | 2.86 | 1.58 | BM124834 |
|  | Transcribed locus | 1435640_x_at | 3.73 | 2.36 | 2.61 | BE634869 |
|  | BB472447 RIKEN full-length enriched, 12 days embryo eyeball Mus musculus cDNA clone D230045E12 3', mRNA sequence. | 1458121_at | 3.73 | 1.87 | 1.96 | BB472447 |
|  | BB134628 RIKEN full-length enriched, adult male bone Mus musculus cDNA clone 9830116N24 3', mRNA sequence. | 1442837_at | 3.39 | 1.76 | 2.52 | BB134628 |
|  | BB667602 RIKEN full-length enriched, adult male liver tumor Mus musculus cDNA clone C730026O19 3', mRNA sequence. | 1443003_at | 3.31 | 2.68 | 2.20 | BB667602 |
|  | AV337434 RIKEN full-length enriched, adult male olfactory bulb Mus musculus cDNA clone 6430400O22 3', mRNA sequence. | 1445402_at | 3.28 | 2.68 | 2.37 | AV337434 |
|  | BB361936 RIKEN full-length enriched, 16 days embryo head Mus musculus cDNA clone C130007L10 3', mRNA sequence. | 1443090_at | 3.01 | 1.63 | 1.76 | BB361936 |
|  | H3044E10-3 NIA Mouse 15K cDNA Clone Set Mus musculus cDNA clone H3044E10 3', mRNA sequence. | 1457851_at | 2.82 | 2.18 | 1.94 | BG066654 |
|  | Transcribed locus, strongly similar to NP_082523.1 carrier family 16 (monocarboxylic acid transporters), member 10 [Mus musculus] | 1440569_at | 2.59 | 1.62 | 2.23 | BB667628 |
|  | BB213784 RIKEN full-length enriched, adult male aorta and vein Mus musculus cDNA clone A530014A13 3', mRNA sequence. | 1443603_at | 2.41 | 2.66 | 1.58 | BB213784 |
|  | Transcribed locus | 1443491_at | 2.41 | 1.74 | 1.58 | BG802688 |
|  | BB282370 RIKEN full-length enriched mouse cDNA library, C57BL/6J retina adult Mus musculus cDNA clone A930037G20 3', mRNA sequence. | 1437600_at | 2.40 | 2.01 | 1.55 | BB282370 |
|  | C0909C05-3 NIA Mouse 12.5-dpc Male Genital Ridge/Mesonephros cDNA Library (Long) Mus musculus cDNA clone C0909C05 3', mRNA sequence. | 1459571_at | 2.28 | 1.61 | 2.22 | BM218317 |
|  | uj45e02.x1 Sugano mouse liver mlia Mus musculus cDNA clone IMAGE:1922906 3', mRNA sequence. | 1440921_at | 2.25 | 2.37 | 2.65 | AI527293 |
|  | BB471757 RIKEN full-length enriched, 12 days embryo eyeball Mus musculus cDNA clone D230040H21 3', mRNA sequence. | 1459608_at | 2.21 | 1.70 | 2.04 | BB471757 |
|  | BB122864 RIKEN full-length enriched, adult male urinary bladder Mus musculus cDNA clone 9530090F02 3', mRNA sequence. | 1441768_at | 2.12 | 1.97 | 1.51 | BB122864 |
|  | BB534083 RIKEN full-length enriched, 0 day neonate lung Mus musculus cDNA clone E030033G19 3', mRNA sequence. | 1442483_at | 2.12 | 1.98 | 1.69 | BB534083 |
|  | AV381845 RIKEN full-length enriched, adult male epididymis Mus musculus cDNA clone 9230119D06 3', mRNA sequence. | 1446503_at | 2.12 | 4.12 | 1.80 | AV381845 |
|  | mu91b02.x1 Soares mouse lymph node NbMLN Mus musculus cDNA clone IMAGE:652875 3', mRNA sequence. | 1457814_at | 2.00 | 6.96 | 1.51 | AI465523 |
|  | C0914H03-3 NIA Mouse 12.5-dpc Male Genital Ridge/Mesonephros cDNA Library (Long) Mus musculus cDNA clone NIA:C0914H03 IMAGE:30035990 3', mRNA sequence. | 1443521_at | 2.00 | 1.51 | 1.62 | BM218716 |
|  | H3075G09-3 NIA Mouse 15K cDNA Clone Set Mus musculus cDNA clone H3075G09 3', mRNA sequence. | 1445001_at | 1.96 | 1.93 | 1.56 | BG069451 |
|  | Transcribed locus | 1444856_at | 1.92 | 2.21 | 1.71 | AW060274 |
|  | Transcribed locus | 1441460_at | 1.91 | 2.16 | 1.56 | BB435465 |
|  | Transcribed locus | 1456156_at | 1.88 | 1.89 | 1.87 | BM124366 |
|  | H3107C12-3 NIA Mouse 15K cDNA Clone Set Mus musculus cDNA clone H3107C12 3', mRNA sequence. | 1445188_at | 1.87 | 2.19 | 1.98 | BG072151 |
|  | Transcribed locus | 1441411_at | 1.86 | 2.44 | 1.71 | BQ031226 |
|  | Transcribed locus | 1437003_at | 1.86 | 2.20 | 1.89 | BB323930 |
|  | BB205199 RIKEN full-length enriched mouse cDNA library, C57BL/6J thymus 0 day neonate Mus musculus cDNA clone A430062L03 3', mRNA sequence. | 1440685_at | 1.86 | 1.73 | 2.90 | BB205199 |
|  | BB485926 RIKEN full-length enriched, 13 days embryo lung Mus musculus cDNA clone D430034A11 3', mRNA sequence. | 1457510_at | 1.83 | 2.01 | 1.70 | BB485926 |
|  | L0922A02-3 NIA Mouse Newborn Kidney cDNA Library (Long) Mus musculus cDNA clone L0922A02 3', mRNA sequence. | 1438265_at | 1.82 | 2.16 | 1.72 | BM119182 |
|  | H3054G06-3 NIA Mouse 15K cDNA Clone Set Mus musculus cDNA clone H3054G06 3', mRNA sequence. | 1457297_at | 1.81 | 2.01 | 1.65 | BG067469 |
|  | Transcribed locus | 1445612_at | 1.80 | 3.69 | 1.73 | AI114898 |
|  | uu57b06.x1 Soares_thymus_2NbMT Mus musculus cDNA clone IMAGE:3376019 3', mRNA sequence. | 1437584_at | 1.79 | 4.19 | 1.67 | BE685667 |
|  | BB629216 RIKEN full-length enriched, 16 days neonate cerebellum Mus musculus cDNA clone 9630059O15 5', mRNA sequence. | 1441131_at | 1.78 | 1.99 | 1.53 | BB629216 |
|  | BB291827 RIKEN full-length enriched, 9.5 days embryo parthenogenote Mus musculus cDNA clone B130008C17 3', mRNA sequence. | 1443012_at | 1.78 | 1.71 | 2.36 | BB291827 |
|  | BB408240 RIKEN full-length enriched, 7 days embryo Mus musculus cDNA clone C430005H19 3', mRNA sequence. | 1437821_at | 1.73 | 2.79 | 1.58 | BB408240 |
|  | K0511D04-3 NIA Mouse Hematopoietic Stem Cell (Lin-/c-Kit+/Sca-1+) cDNA Library (Long) Mus musculus cDNA clone NIA:K0511D04 IMAGE:30064935 3', mRNA sequence. | 1456715_at | 1.70 | 2.24 | 16.41 | BM238052 |
|  | BB725729 RIKEN full-length enriched, 8 cells embryo Mus musculus cDNA clone E860007B07 3', mRNA sequence. | 1447381_at | 1.70 | 1.53 | 2.15 | BB725729 |
|  | BB490576 RIKEN full-length enriched, 13 days embryo stomach Mus musculus cDNA clone D530016G24 3', mRNA sequence. | 1441365_at | 1.68 | 1.56 | 10.61 | BB490576 |
|  | Transcribed locus | 1441660_at | 1.67 | 1.94 | 2.05 | AV351863 |
|  | K0545A08-3 NIA Mouse Hematopoietic Stem Cell (Lin-/c-Kit+/Sca-1+) cDNA Library (Long) Mus musculus cDNA clone NIA:K0545A08 IMAGE:30068167 3', mRNA sequence. | 1439301_at | 1.67 | 4.80 | 2.84 | BM240022 |
|  | BB053697 RIKEN full-length enriched, 12 days embryo male wolffian duct Mus musculus cDNA clone 6720465D13 3', mRNA sequence. | 1446068_at | 1.65 | 2.19 | 1.59 | BB053697 |
|  | BB054169 RIKEN full-length enriched mouse cDNA library, C57BL/6J wolffian duct includes surrounding region male 12 days embryo Mus musculus cDNA clone 6720469B08 3', mRNA sequence. | 1439307_at | 1.62 | 1.72 | 1.91 | BB054169 |
|  | Transcribed locus | 1460126_at | 1.62 | 1.61 | 1.64 | BG070566 |
|  | BB210636 RIKEN full-length enriched, 0 day neonate thymus Mus musculus cDNA clone A430098L10 3', mRNA sequence. | 1458610_at | 1.61 | 1.61 | 1.69 | BB210636 |
|  | H3071C09-3 NIA Mouse 15K cDNA Clone Set Mus musculus cDNA clone H3071C09 3', mRNA sequence. | 1442381_at | 1.59 | 1.69 | 2.53 | BG068971 |
|  | C78040 Mouse 3.5-dpc blastocyst cDNA Mus musculus cDNA clone J0041H03 3', mRNA sequence. | 1445512_at | 1.59 | 1.58 | 2.09 | C78040 |
|  | BB283832 RIKEN full-length enriched, adult retina Mus musculus cDNA clone A930103I05 3', mRNA sequence. | 1445562_at | 1.55 | 3.20 | 2.54 | BB283832 |
|  | BB214530 RIKEN full-length enriched, adult male aorta and vein Mus musculus cDNA clone A530021B03 3', mRNA sequence. | 1447116_at | 1.53 | 5.52 | 2.73 | BB214530 |
|  | BB107628 RIKEN full-length enriched, adult male urinary bladder Mus musculus cDNA clone 9530006N05 3', mRNA sequence. | 1443949_at | 1.53 | 1.99 | 1.66 | BB107628 |
|  | Transcribed locus | 1457259_at | 1.52 | 1.65 | 1.76 | AV023631 |
|  | K0154C12-3 NIA Mouse Hematopoietic Stem Cell (Lin-/c-Kit+/Sca-1-) cDNA Library (Long) Mus musculus cDNA clone NIA:K0154C12 IMAGE:30044579 3', mRNA sequence. | 1440040_at | 1.51 | 1.73 | 2.47 | BM224656 |
