# Supplementary material for: Altered Energy Homeostasis and Resistance to Diet-Induced Obesity in KRAP-Deficient Mice
Source: PLoS One. 2009 Jan 21;4(1):e4240. doi: 10.1371/journal.pone.0004240 (PMC2627767; doi:10.1371/journal.pone.0004240)
Supplement: Table S5 — Primer sequences for preparation of probes used in northern blotting. (0.03 MB DOC) [file pone.0004240.s011.doc]

| **Symbol/Gene name** | **5' primer** | **3' primer** | **ACCESSION** |
| --- | --- | --- | --- |
| *Acc1* | 5'-ggggcggccgcgtctttgccaactggagagg | 5'-ggggcggccgccgtggaaggggaatccattg | AY451393 |
| *Acc2* | 5'-ggggcggccgcctcatgatcttcgccaactg | 5'-ggggcggccgcggaggaagctgggctctccg | AY451394 |
| *Acox1* | 5'-cgcactgccactacgtgaccgtta | 5'-tcaaagcttcgactgcaggggct | AF006688 |
| *Ucp2* | 5'-ggggcggccgcatggttggtttcaaggccac | 5'-ggggcggccgctcagaaaggtgcctcccgag | NM_011671 |
| *Actb* | 5'-atggatgacgatatcgctgcg | 5'-gaagctgtagccacgctcgg | NM_007393 |
| *Lpl* | 5'-ggggcggccgcatggagagcaaagccctgct | 5'-ggggcggccgcgagcgagtcttcaggtacat | NM_008509 |
| *Hsl* | 5'-ggggcggccgcgtgggaatctctgcatcact | 5'-ggggcggccgctcagttcagtggtgcagcagg | BC021642 |
| *Pparg* | 5'-ggggcggccgcatgggtgaaactctgggaga | 5'-ggggcggccgcatactctgtgatctcttgca | NM_011146 |
| *Leptin* | 5'-ggggcggccgcatgtgctggagacccctgtg | 5'-ggggcggccgctcagcattcagggctaacat | NM_008493 |
| *Fabp4* | 5'-ggggcggccgcatgtgtgatgcctttgtggg | 5'-ggggcggccgcatccgactgactattgtagt | NM_024406 |
| *Rplp0* | 5'-ggggcggccgcatgcccagggaagacagggc | 5'-ggggcggccgcgtcgaagagaccgaatccca | NM_007475 |
| *Ucp1* | 5'-ggggcggccgcatggtgaacccgacaacttc | 5'-ggggcggccgcttatgtggtacaatccactg | NM_009463 |
| *Pgc1a* | 5'-ggggcggccgcatggcttgggacatgtgca | 5'-ggggcggccgcgtagtttggagaattgttca | AF049330 |
