# Supplementary material for: Altered Energy Homeostasis and Resistance to Diet-Induced Obesity in KRAP-Deficient Mice
Source: PLoS One. 2009 Jan 21;4(1):e4240. doi: 10.1371/journal.pone.0004240 (PMC2627767; doi:10.1371/journal.pone.0004240)
Supplement: Supplementary file 6 [file pone.0004240.s007.doc]

Parameters	meal	units	n	wild-type	$KRAP^{-/-}$	P-value
Weight at birth	—	g	14	1.36 ± 0.04	1.38 ± 0.04	0.60
Liver glycogen content	standard diet	g/g-tissue	9	55.8 ± 6.3	51.5 ± 6.9	0.66
Protein content in feces	standard diet	mg/g-feces	10	45.9 ± 6.0	42.9 ± 4.6	0.70
Respiratory quotient	standard diet	—	7-9	0.90 ± 0.03	0.88 ± 0.02	0.37
Respiratory quotient	high-fat diet	—	5-6	0.85 ± 0.02	0.86 ± 0.01	0.37
Liver triglyceride content	high-fat diet	mg/g-tissue	9	280.1 ± 20.4	17.4 ± 1.4	< 0.001
Muscle triglyceride content	high-fat diet	mg/g-tissue	8	106.0 ± 17.7	39.3 ± 10.2	< 0.001
Serum glucose	high-fat diet	mg/dl	7-8	228 ± 20	194 ± 8	0.15
Serum insulin	high-fat diet	ng/ml	7-8	13.5 ± 1.8	5.6 ± 0.9	0.003
Serum leptin	high-fat diet	ng/ml	7-8	25.2 ± 1.5	13.4 ± 2.7	0.003
